# Characterization of Gram-negative Bloodstream Infections in Hospitalized Australian Children and Their Clinical Outcomes

**DOI:** 10.1093/cid/ciae341

**Published:** 2024-06-25

**Authors:** Sophie C H Wen, Patrick N A Harris, Brian Forde, Budi Permana, Mark D Chatfield, Colleen L Lau, Geoffrey Spurling, Michelle J Bauer, Ross Balch, Henry Chambers, Luregn J Schlapbach, Julia E Clark, Sonia Dougherty, Christopher C Blyth, Philip N Britton, Vanessa Clifford, Gabrielle M Haeusler, Brendan McMullan, Ushma Wadia, David L Paterson, Adam D Irwin

**Affiliations:** The University of Queensland, Centre for Clinical Research, Brisbane, Queensland, Australia; Infection Management Prevention Service, Children's Health Queensland, Brisbane, Queensland, Australia; The University of Queensland, Centre for Clinical Research, Brisbane, Queensland, Australia; The University of Queensland, Centre for Clinical Research, Brisbane, Queensland, Australia; The University of Queensland, Centre for Clinical Research, Brisbane, Queensland, Australia; Herston Infectious Diseases Institute, Metro North Health, Brisbane, Queensland, Australia; The University of Queensland, Centre for Clinical Research, Brisbane, Queensland, Australia; The University of Queensland, Centre for Clinical Research, Brisbane, Queensland, Australia; The University of Queensland, General Practice Clinical Unit, Brisbane, Queensland, Australia; The University of Queensland, Centre for Clinical Research, Brisbane, Queensland, Australia; The University of Queensland, Centre for Clinical Research, Brisbane, Queensland, Australia; School of Medicine, University of California San Francisco, San Francisco, California, USA; The University of Queensland, Child Health Research Centre, Brisbane, Queensland, Australia; Infection Management Prevention Service, Children's Health Queensland, Brisbane, Queensland, Australia; Infection Management Prevention Service, Children's Health Queensland, Brisbane, Queensland, Australia; Wesfarmer Centre of Vaccines and Infectious Diseases, Telethon Kids Institute, Perth, Western Australia, Australia; Department of Infectious Diseases, Perth Children's Hospital, Perth, Western Australia, Australia; Department of Microbiology, PathWest Laboratory Network, Perth, Western Australia, Australia; School of Medicine, University of Western Australia, Perth, Western Australia, Australia; Department of Infectious Diseases and Microbiology, The Children's Hospital Westmead, Sydney, New South Wales, Australia; Sydney Medical School and Sydney Infectious Diseases, University of Sydney, Sydney, New South Wales, Australia; Infectious Diseases Unit, Royal Children's Hospital, Melbourne, Victoria, Australia; Infectious Diseases Unit, Royal Children's Hospital, Melbourne, Victoria, Australia; Clinical Infections Group, Murdoch Children's Research Institute, Melbourne, Victoria, Australia; Infectious Diseases and Microbiology, Sydney Children's Hospital, Sydney, New South Wales, Australia; Faculty of Medicine and Health, University of New South Wales, Sydney, New South Wales, Australia; Wesfarmer Centre of Vaccines and Infectious Diseases, Telethon Kids Institute, Perth, Western Australia, Australia; Department of Infectious Diseases, Perth Children's Hospital, Perth, Western Australia, Australia; School of Medicine, University of Western Australia, Perth, Western Australia, Australia; ADVANCE-ID, Saw Swee Hock School of Public Health, National University of Singapore, Singapore; Infectious Diseases Translational Research Programme, Yong Loo Lin School of Medicine, National University of Singapore, Singapore; The University of Queensland, Centre for Clinical Research, Brisbane, Queensland, Australia; Infection Management Prevention Service, Children's Health Queensland, Brisbane, Queensland, Australia

**Keywords:** Gram-negative, pediatric, bloodstream infections, antimicrobial resistance, outcome

## Abstract

**Background:**

Gram-negative bloodstream infections (GNBSIs) more commonly occur in children with comorbidities and are increasingly associated with antimicrobial resistance. There are few large studies of GNBSIs in children that relate the clinical presentation, pathogen characteristics, and outcomes.

**Methods:**

A 3-year prospective study of GNBSIs in children aged <18 years was conducted in 5 Australian children's hospitals between 2019 and 2021. The clinical characteristics, disease severity, and outcomes were recorded. Causative pathogens underwent antibiotic susceptibility testing and whole genome sequencing.

**Results:**

There were 931 GNBSI episodes involving 818 children. Median age was 3 years (interquartile range, 0.6–8.5). A total of 576/931 episodes (62%) were community onset, though 661/931 (71%) occurred in children with comorbidities and a central venous catheter was present in 558/931 (60%). Central venous catheter (145/931) and urinary tract (149/931) were the most common sources (16% each). One hundred of 931 (11%) children required intensive care unit admission and a further 11% (105/931) developed GNBSIs in intensive care unit. A total of 659/927 (71%) isolates were Enterobacterales, of which 22% (138/630) were third-generation cephalosporin resistant (3GCR). Extended spectrum beta-lactamase genes were confirmed in 65/138 (47%) 3GCR Enterobacterales. Most common extended spectrum beta-lactamase genes were *bla*_CTX-M-15_ (34/94, 36%) and *bla*_SHV-12_ (10/94, 11%). There were 48 deaths overall and 30-day in-hospital mortality was 3% (32/931). Infections with 3GCR Enterobacterales were independently associated with higher mortality (adjusted odds ratio, 3.2; 95% confidence interval, 1.6–6.4).

**Conclusions:**

GNBSIs in children are frequently healthcare associated and affect children younger than age 5 years. Infections with 3GCR Enterobacterales were associated with worse outcomes. These findings will inform optimal management guidelines and help prioritize future antimicrobial clinical trials.

## Teaser

A total of 931 gram-negative bloodstream infection episodes involving 818 children were studied. The children were typically young and medically complex. Enterobacterales comprised 71%, of which 22% demonstrated third-generation cephalosporin resistance. Bloodstream infections caused by Enterobacterales resistant to third-generation cephalosporin were associated with worse outcomes.

## Graphical Abstract

**Graphical Abstract:**
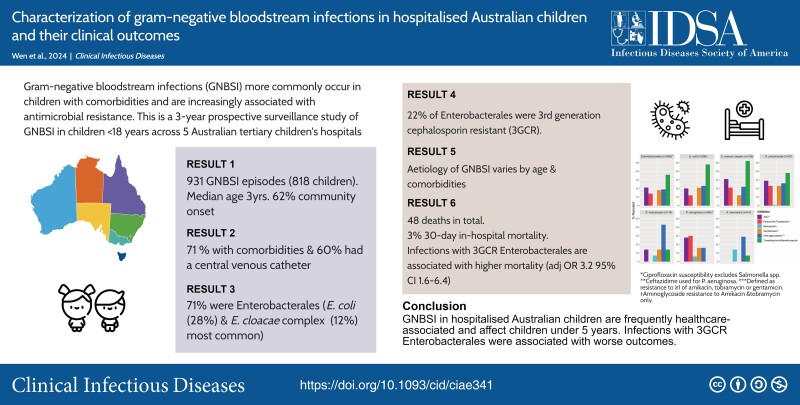
This graphical abstract is also available at Tidbit: https://tidbitapp.io/institutional-portal/clinical-infectious-diseases/tidbits/characterization-of-gram-negative-bloodstream-infections-in-hospitalised-australian-children-and-their-clinical-outcomes/update

## BACKGROUND

Gram-negative bloodstream infections (GNBSIs) in children are associated with significant morbidity and mortality. Their management is complicated by a growing burden of antimicrobial resistance (AMR), particularly in neonates [1–3]. Despite the importance of these infections, there are comparatively few studies that have systematically described the clinical and molecular epidemiology of GNBSIs in children [4, 5]. These studies have been limited to single centers [6], focused on specific clinical contexts, such as children with cancer [7], or on infection types such as central venous catheter (CVC)-associated infections [8, 9]. Clinical outcomes following GNBSIs in children have not been well described, particularly in association with AMR in a high-income setting; therefore, the risk factors associated with adverse outcomes remain poorly understood.

The optimal management of resistant GNBSIs in children is unclear, and guidance is drawn largely from adult studies. Unlicensed and off-label prescribing of antimicrobials is common in children, often because of a lack of relevant antimicrobial clinical trials [10]. There is a marked disparity between antibiotic development and research programs in adults and children [11], resulting in a lag of up to 10 years before a newly available drug for adults is tailored for use in children.

To design quality antimicrobial clinical trials for GNBSIs in children, a better understanding of the host and pathogen characteristics associated with adverse outcomes is required. We therefore conducted a 3-year, prospective, multicenter surveillance study of GNBSIs in hospitalized Australian children. The primary objectives were to systematically characterize the pathogens causing GNBSIs in children and to identify risk factors for adverse outcomes.

## METHODS

### Study Design

This prospective surveillance study of GNBSIs in hospitalized Australian children was undertaken through the national Paediatric Active Enhanced Disease Surveillance network (www.paeds.org.au) [12] between January 2019 and December 2021. Participants were recruited from 5 tertiary pediatric hospitals across Australia.

Children aged <18 years with GNBSIs, defined according to the Australian Commission on Safety and Quality in Healthcare (Supplementary section 1), were included. Interhospital transfers were included if they had a positive culture at the study site. Environmental gram-negative organisms and other potential contaminants were included if cultured on 2 separate occasions within a 48-hour period.

Demographics, comorbidities, infection source, organisms and antimicrobial susceptibilities, disease severity, and outcomes were collected from hospital and laboratory records and entered into a REDCap database hosted at the University of Queensland [13, 14].

Significant comorbidities were defined as chronic medical conditions expected to persist beyond 12 months that require specialist pediatric input and categorized according to the Pediatric Complex Chronic Classification system version 2 [15]. Community- and hospital-onset GNBSIs were defined as a positive blood culture collected ≤48 hours or >48 hours after hospital presentation, respectively. Among community-onset infections, the National Healthcare Safety Network Centres for Disease Control and Prevention definitions of healthcare-associated infections were used [16].

To calculate the duration of hospitalization for participants admitted for other reasons, the start date was defined as the date of first positive blood culture with a gram-negative pathogen(s). The end date was defined as date of hospital discharge or death. All-cause, 30-day, 90-day, and GNBSI-attributable mortality were collected for in-hospital deaths.

### Microbiological Methods

All blood cultures were processed using standard commercial blood culture systems (BACT/ALERT VIRTUO, bioMérieux, France), bacterial identification (matrix-assisted laser desorption/ionization time-of-flight; Vitek MS, bioMérieux), and semiautomated susceptibility platforms (Vitek 2, bioMérieux). Gram-negative isolates were shipped to a research laboratory at the University of Queensland Centre for Clinical Research for additional broth microdilution susceptibility testing (Sensititre; Thermo Fisher Scientific). Minimum inhibitory concentrations were interpreted using European Committee on Antimicrobial Susceptibility Testing clinical breakpoints [17]. In Enterobacterales, we used third-generation cephalosporin (3GC) resistance (ceftriaxone minimum inhibitory concentration >2 mg/L or ceftazidime >4 mg/L) to indicate the presence of either extended-spectrum beta-lactamase (ESBL) or other cephalosporinases (eg, AmpC). Multidrug resistance (MDR) was defined as resistance to ≥1 agent in ≥3 antimicrobial categories [18] (full details in Supplementary section 2).

### Whole Genome Sequencing

Whole genome sequencing (WGS) of isolates was performed using the Illumina NextSeq 500 platform with 150 base-pair paired-end chemistry. Genomic analysis was performed using a custom, in-house microbial genomic analysis pipeline, SnapperRocks (https://github.com/FordeGenomics/SnapperRocks) [19]. In brief, the quality of reads was checked using FastQC v0.11.6 [20] and low-quality reads and adapters were trimmed using Trimmomatic v0.36 [21]. Contamination detection and taxonomic classification were performed using Kraken v.2.0.8 [22]. De novo assembly was performed using SPAdes v3.14.0 [23]. In silico multilocus sequence typing was performed using srst2 v0.20 [24] and abricate v0.9.8 (https://github.com/tseemann/abricate). In silico antimicrobial resistance genes were identified using AMRFinderPlus v3.10 [25].

### Statistical Analysis

The χ^2^ test or Fisher exact test was used to compare categorical variables and the Student *t* test or Mann-Whitney *U* test was used to compare continuous variables. *P* values <.05 were considered statistically significant. Univariate logistic regression was used to explore potential determinants of death. Variables with *P* value <.2 from univariate logistic regression were combined with variables selected based on subject matter expertise to develop a multivariable model (ie, age, presence of CVC, or comorbidities). A maximal model was first constructed before variables (other than those based on subject matter expertise) were removed in a backwards stepwise fashion if no significant differences in fit was observed. Statistical analyses were performed using Stata 17 [26].

The study received ethical approval from the Sydney Children's Hospital Network Human Research Ethics Committee (HREC/18/SCHN/72) and local governance approvals at participating sites.

## RESULTS

### Epidemiology and Clinical Characteristics

There were 931 episodes of GNBSIs reported in 818 children. The overall incidence of GNBSIs was 200/100 000 hospitalizations (95% confidence interval [CI], 188–214). Site-specific incidences are summarized in Supplementary section 3. The characteristics and distribution of cases are summarized in Table 1. The median age was 3 years (interquartile range [IQR], 0.6–8.5) with 10% (91/931) in neonates <28 days and 21% (197/931) in infants 28 days to 1 year. A total of 58% (539/931) occurred in males, 6% (60/931) were Aboriginal and or Torres Strait Islander children and 9% (83/931) were born outside of Australia. Overall, 62% (576/931) were community-onset infections; however, site-specific differences were observed (Supplementary section 3). A significant underlying comorbidity was documented in 71% (661/931) of episodes, and 24% had ≥2 comorbidities. The most common comorbidity was malignancy (37%). The presence of a CVC was the most common healthcare-associated risk factor (60%). Antimicrobial therapy was highly variable, even within species (see Supplementary materials, section 10).

**Table 1.** Characteristics of Children in 931 GNBSI Episodes Across 5 Tertiary Pediatric Hospitals in Australia, 2019–2021

Characteristics	N (%)
Total	931
Gender	
Female	392 (42)
Male	539 (58)
Age	
Neonates (<28 d)	91 (10
Infants (28–364 d)	197 (21)
1–4 y	285 (31)
5–9 y	164 (18)
10–14 y	115 (12)
15–17 y	75 (8)
Missing	4 (0)
Ethnicity	
Aboriginal and/or Torres Strait Islander	60 (6)
Non-Aboriginal or Torres Strait Islander	865 (93)
Missing	6 (1)
Country of birth	
Australia	783 (84)
Other	83 (9)
Unknown	65 (7)
Location	
Site 1	221 (24)
Site 2	71 (8)
Site 3	249 (27)
Site 4	300 (32)
Site 5	90 (10)
Comorbidities	
None	270 (29)
1	434 (47)
2	150 (16)
3	50 (5)
≥4	27 (3)
Types of comorbidities	
Malignancy	343 (37)
Hematologic/immunologic	146 (16)
Congenital/genetic	100 (11)
Gastrointestinal	98 (11)
Cardiovascular	78 (8)
Neurological	79 (8)
Renal	52 (6)
Respiratory	46 (5)
Neonatal	38 (4)
Metabolic	25 (3)
Setting	
Community onset	576 (62)
Healthcare associated	272/576 (47)
Hospital onset	350 (38)
ICU onset	105/350 (30)
Missing	5 (0)
Healthcare-associated factors^a^	
Indwelling CVC	558 (60)
Other indwelling device/non-native material	179 (19)
Receiving TPN	122 (13)
Surgery within past 30 d^b^	238 (26)
Neutropenia	309 (33)
Focus of infection	
None identified	343 (37)
Intravascular device	145 (16)
Organ specific	442 (47)
Urinary tract	149 (34)
Intra-abdominal	112 (25)
Respiratory tract	31 (7)
Mucosal barrier injury	52 (12)
Central nervous system	11 (2)
Other	87 (20)
Missing	1 (0)

Abbreviations: CVC, central venous catheter; GNBSI, Gram-negative bloodstream infections; ICU, intensive care unit; TPN, total parenteral nutrition.

^a^Multiple factors possible.

^b^Details of surgical procedure in Supplementary section 9.

The focus of GNBSIs was identified in 63% (587/930); with 16% attributed to a CVC. An organ-specific focus was reported in 48% (442/930), with urinary tract being the most common (16%, Table 1).

Fever was present at onset of GNBSIs in 85% (791/931) of episodes with a median duration of 2 days (IQR, 1–4 days). An elevated C-reactive protein (CRP, >10 mg/L) was documented in 88% (550/623). Median peak CRP during GNBSIs was 90 mg/L (IQR, 49–17) and the median duration of abnormal CRP was 13 days (IQR, 4–30).

Pediatric intensive care unit (PICU) admission for GNBSIs was required in 11% (100/931), whereas 11% (105/931) were already in the PICU at the time of GNBSIs. Intensive care supports required during PICU admission are detailed in Table 2.

**Table 2.** Outcomes of (1) the 931 GNBSI Episodes, (2) the 138 Episodes With 3GC Resistant Enterobacterales BSI, and (3) the 486 Episodes With 3GC Susceptible Enterobacterales BSI in Children Across 5 Australian Tertiary Pediatric Hospitals, 2019–2021

Outcomes	Total (N = 931)	3GC-resistant Enterobacterales BSI (N = 138)	3GC-susceptible Enterobacterales BSI (N = 486)	*P* Value^a^
Relapse within 14 d	25 (3%)	1 (1%)	12 (2%)	.21
PICU admission required for GNBSI (onset out of PICU)	100 (11%)	12 (9%)	51 (11%)	.74
Onset of GNBSI in PICU	105 (11%)	24 (17%)	48 (10%)	.015
Invasive ventilation	103 (11%)	22 (16%)	46 (9%)	.043
Inotropic support	88 (9%)	16 (12%)	47 (10%)	.52
Renal replacement therapy	9 (10%)	3 (2%)	4 (1%)	.19
Mortality				
All-cause deaths	48 (5%)	17 (12%)	18 (4%)	<.001
GNBSI-related deaths	29 (3%)	11 (8%)	9 (2%)	<.001
30-d in-hospital mortality	32 (3%)	11 (8%)	11 (2%)	.001
90-d in-hospital mortality	44 (5%)	16 (12%)	16 (3%)	<.001
Length of stay				
Duration of hospitalization overall	12 (7–24)	17 (10–33)	12 (7–21)	<.001
Duration of PICU admission overall^b^	8 (2–25)	12 (2.5–40)	5 (2–18)	.035
Duration of PICU admission for onset of GNBSI out of PICU	3 (1–7)	3.5 (2–18.5)	2.5 (1–7)	.22

Data are presented as median days (IQR) for continuous variables and number (%) for categorical/binary variables.

Abbreviations: 3GC, third-generation cephalosporin; BSI, bloodstream infection; GNBSI, gram-negative bloodstream infection; PICU, pediatric intensive care unit.

^a^Comparison between 3GC-resistant and susceptible Enterobacterales bloodstream infections.

^b^Includes admissions that were not initially from GNBSI.

### Microbiology and Antimicrobial Resistance

A total of 1021 pathogens were isolated from 931 episodes; 927 isolates were identified to species level and antimicrobial susceptibility data were available for 852; and 93% of episodes had a single organism isolated. Enterobacterales accounted for 71% of gram-negative isolates identified.

A total of 89 species, representing 34 genera, were identified by WGS (n = 888) including *Escherichia coli* (n = 253), *Enterobacter* species (n = 116), *Salmonella enterica* (n = 94), *Klebsiella pneumoniae* (n = 83), *Pseudomonas aeruginosa* (n = 81), *Stenotrophomonas maltophilia* (n = 23), *Serratia marcescens* (n = 16), and *Acinetobacter baumannii* (n = 13). These 8 species accounted for 76% of isolates, with the remaining 209 isolates distributed among 70 different species. Significant intraspecies diversity was observed among the 8 most abundant sequenced isolates, with a total of 249 distinct lineages identified (Table 3).

**Table 3.** Lineage Diversity Among Sequenced Isolates (n = 888) in Children With GNBSI Across 5 Tertiary Pediatric Hospitals in Australia: 2019–2021

Species	No. of Isolates	No. of STs	No. of Isolates With Uncharacterized STs^b^
*E. coli*	253	61	1
*Enterobacter* spp.	116	46	19
*S. enterica*	94	26	0
*K. pneumoniae*	83	54	4
*P. aeruginosa*	81	42	8
*S. maltophilia*	23	12	7
*S. marcescens*	16	NA^a^	16
*A. baumannii*	13	8	2
Other^c^	209	-	-

Abbreviations: GNBSI, gram-negative bloodstream infection; MLST, multilocus sequence type; NA, not applicable; ST, sequence type.

^a^No MLST profile defined.

^b^Isolates may belong to the same uncharacterized ST.

^c^This included 72 species.

**Figure 1.**
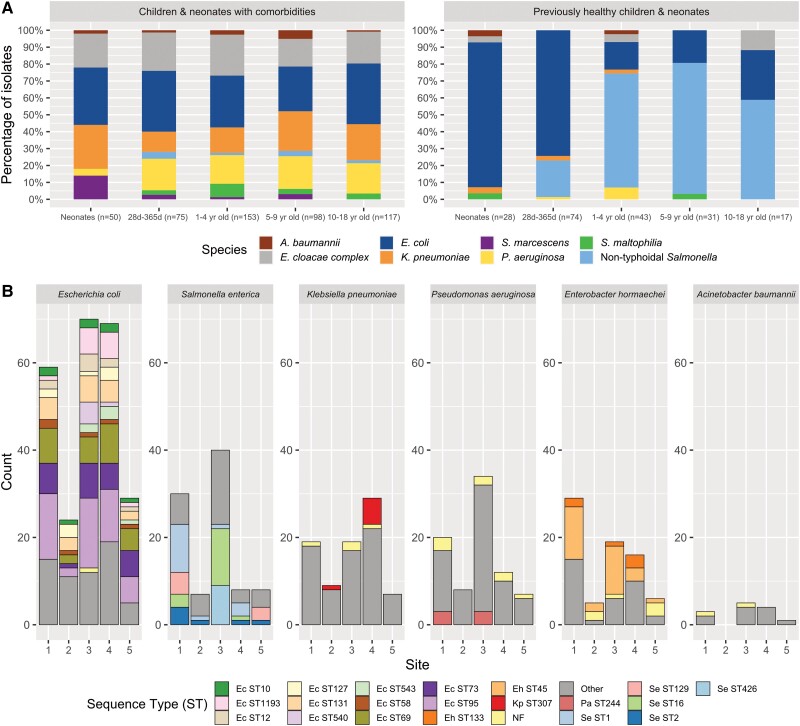
Microbiology of gram-negative bloodstream infections (GNBSIs) in children. *A*, Distribution of gram-negative bacteria isolated in blood cultures based on age group and comorbidities. *B*, Distribution of key gram-negative bacteria and sequence types across participating sites.

Figure 1 *A* illustrates the distribution of organisms by age and comorbidities. *E. coli* was most common in children aged <1 year (43%, 123/286) and in neonates (42%, 41/97). Significant age differences were observed in *E. coli*, *K. pneumoniae*, nontyphoidal *Salmonella* spp., *P. aeruginosa*, and *S. marcescens*. In children with comorbidities, *K. pneumoniae*, *P. aeruginosa*, *S. marcescens*, *S. maltophilia*, and *E. cloacae* complex (ECC) occurred more commonly (*P* < .05), whereas *E.coli* and nontyphoidal *Salmonella* spp. were more common in children without comorbidities (*P* < .001, Supplementary section 4). Six infections with *P. aeruginosa* or *A. baumannii* were observed in previously healthy children.

The most common organisms isolated from CVC-associated infections were *Klebsiella* spp. (27%, 39/145), ECC (23%, 33/145), and *Pseudomonas* spp. (17%, 24/145). The most common organisms in urinary tract and intra-abdominal infections were *E. coli* (77%, 115/149) and *Salmonella* spp. (37%, 46/126), respectively.

The distribution of the 6 most abundant species (*E. coli*, *S. enterica*, *K. pneumoniae*, *P. aeruginosa*, *Enterobacter hormaechei*, and *A. baumannii*) and associated sequence type (ST) by hospital are illustrated in Figure 1*B*. ST distribution was significantly different between sites for *K. pneumoniae*, *S. enterica*, *P. aeruginosa*, and *E. hormaechei* (*P* < .05, Supplementary section 5). The 5 most common *E. coli* STs were ST95 (20%), ST69 (12%), ST73 (11%), ST131 (8%), and ST1193 (6%). ST95 accounted for 33% of *E. coli* urinary tract infections. There were no significant differences in *E. coli* ST distribution between age groups, presence of comorbidity, or mortality (Supplementary section 5). For *K. pneumoniae*, the most common STs were ST307 (8%), ST17 (5%), and ST34 (5%). ST307 was the only major AMR-related *K. pneumoniae* ST identified.

ESBL genes were detected in 108 isolates, of which 87% (94/108) were Enterobacterales. The most common ESBL genes identified in Enterobacterales were *bla*_CTX-M-15_ (36%, 34/94) and *bla*_SHV-12_ (11%, 10/94). ESBL genes were more commonly detected in *K. pneumoniae* (27%, 22/82) than in *E. coli* (12%, 29/246) and ECC (10%, 11/114). The dominant ESBL gene carrying STs in *E. coli* were ST131 (21%, 6/29), ST69 (17%, 5/29), and ST1193 (14%, 4/29). Phenotypic 3GC resistance (3GCR) was observed in 22% (138/630) of all Enterobacterales and ESBL genes were confirmed in 47% of these (65/138). Of the 73 non-ESBL 3GCR-Enterobacterales, 35 isolates (48%) had chromosomally encoded AmpC genes (2 *Citrobacter braakii*, 1 *C. freundii*, 24 *Enterobacter* spp., 1. *K. aerogenes*, and 2 *S. marcescens*). Plasmid-mediated AmpC genes detected include *bla*_DHA1_ (1 *E. coli*, 3 *K. pneumoniae*) and *bla*_CMY-42_ (3 *E. coli* isolates). Overall, 34% of Enterobacterales were MDR (Supplementary section 2), with 19% aminoglycoside resistance (detailed aminoglycoside resistance data in Supplementary section 6).

Phenotypic meropenem resistance was only observed in 0.5% (3/630) of Enterobacterales (1 *E. coli*, 1 *K. pneumoniae*, 1 *S. marcescens*); no carbapenemase genes were detected in these 3 isolates. Carbapenemase genes and porin mutations were detected in 9 Enterobacterales isolates that did not exhibit phenotypic resistance (4 *K. pneumoniae*, 4 *E. cloacae complex*, 1 *K. variicola*), most commonly *bla*_IMP-4_ (44%, 4/9) and blaNDM-1 (11%, 1/9). Major outer membrane porin mutations, ompk35_E132 K were detected only in 4 *Klebsiella* isolates. Two *Acinetobacter* spp. isolates were found to be phenotypically resistant to carbapenems (carrying *bla*_OXA-58_ and *bla*_OXA-500_).

**Figure 2.**
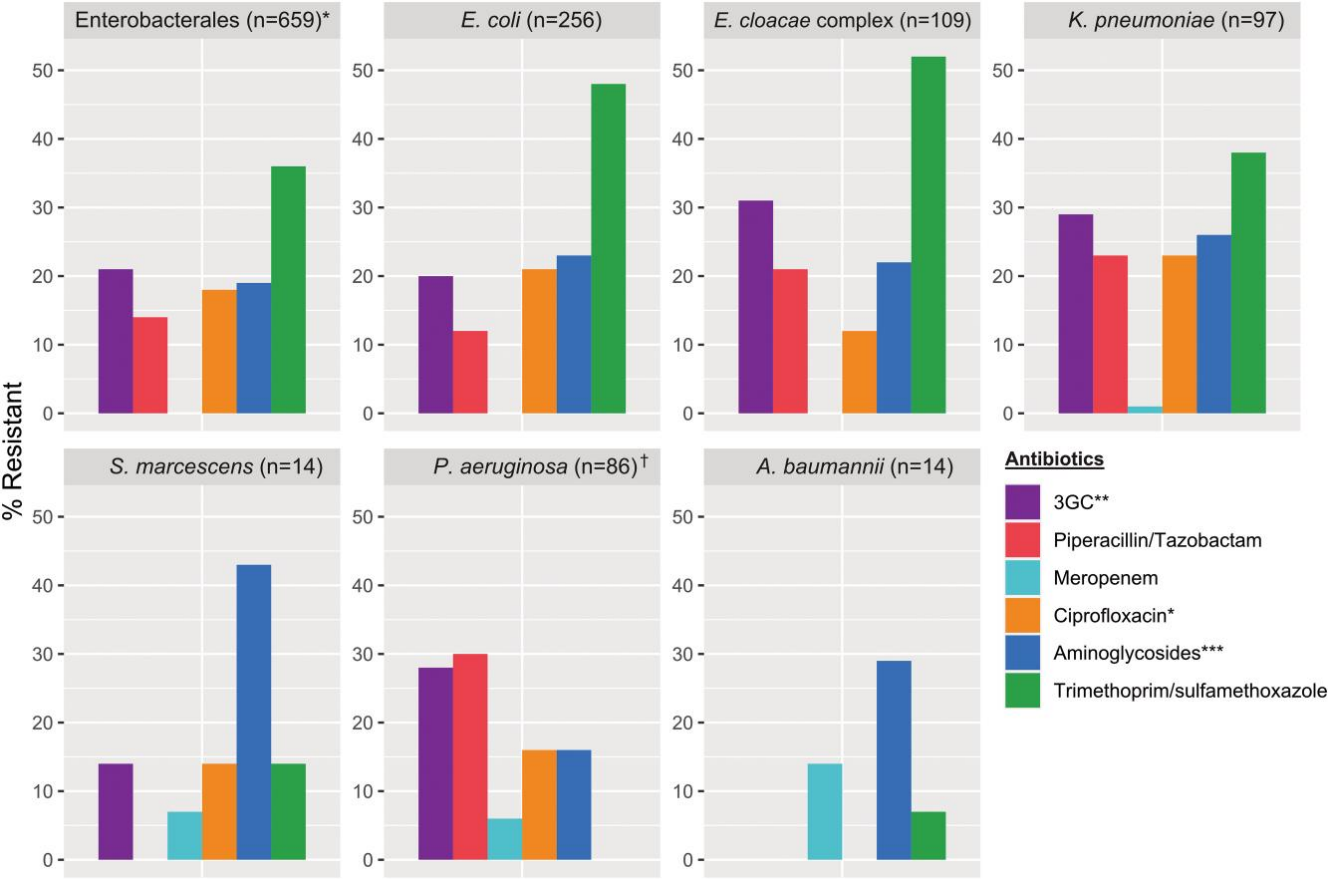
Antimicrobial resistance patterns of gram-negative bloodstream infections (GNBSIs) in children across 5 Australian tertiary pediatric hospitals, 2019–2021. 3GC, third-generation cephalosporin. *Ciprofloxacin susceptibility excludes *Salmonella* spp. **Ceftazidime used for *P. aeruginosa*. ***Defined as resistance to ≥1 of amikacin, tobramycin, or gentamicin. †Aminoglycoside resistance to amikacin and tobramycin only.

**Figure 3.**
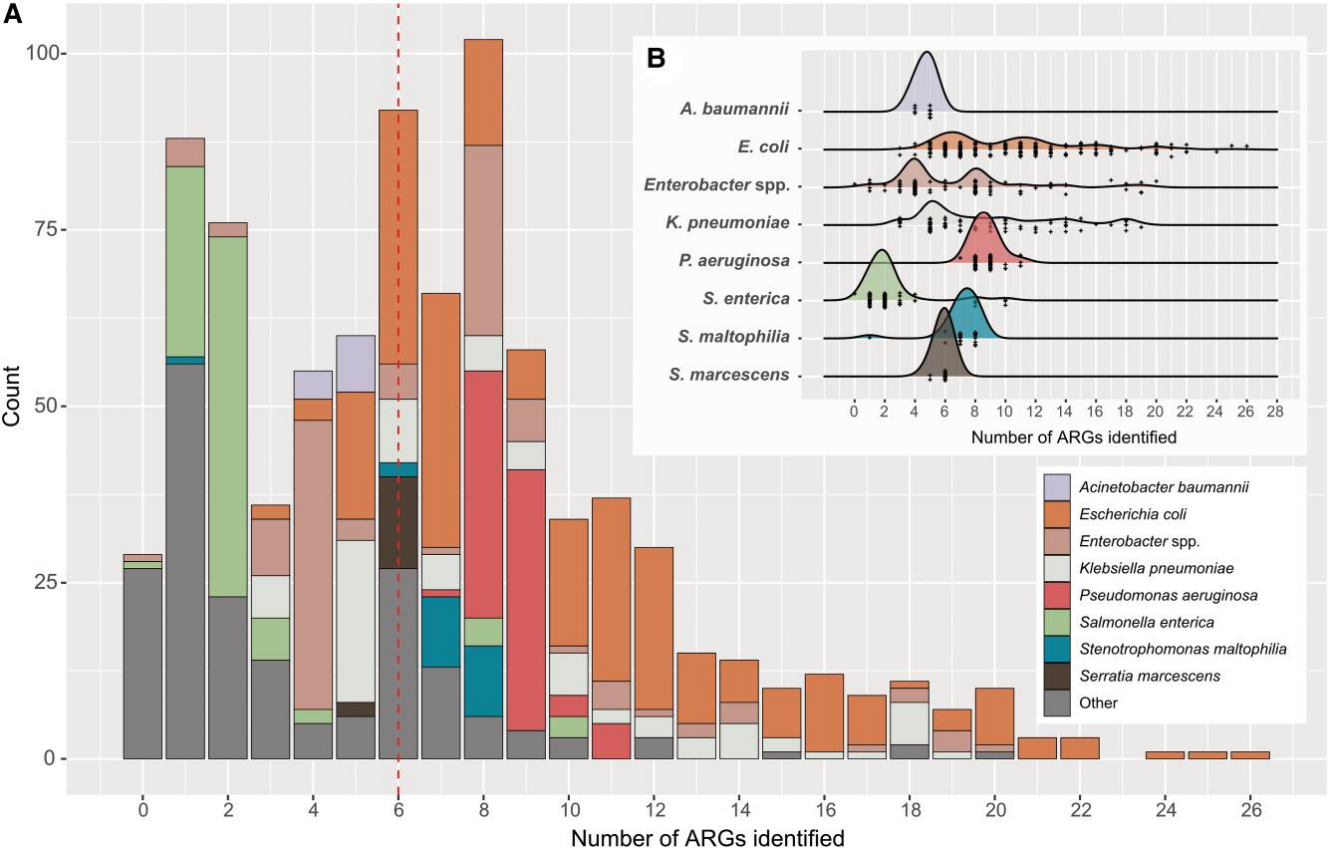
Antimicrobial resistance gene carriage in all sequenced gram-negative isolates (N = 880) from children with GNBSIs across 5 tertiary pediatric hospitals in Australia, 2019–2021. *A*, Frequency distribution of ARG carriage in all sequenced isolates. Red dashed line represents the median (n = 6) number of ARG identified in sequenced genomes. *B*, Ridge plots highlighting intraspecies diversity of ARG carriage among the most frequently identified clinical isolates. ARG, antimicrobial resistance gene; GNBSI, gram-negative bloodstream infection.

Sulfamethoxazole/trimethoprim resistance was noted in 14% (3/22) of *S. maltophilia*. Figure 2 summarizes antimicrobial resistance in Enterobacterales, *A. baumannii*, and *P. aeruginosa*. Resistance rates to ceftolozane/tazobactam and ceftazidime/avibactam in *P. aeruginosa* were 19% (16/86) and 17% (15/86), respectively. There was substantial intra- and interspecies variability in carriage of AMR genes (median number of AMR genes = 6; range, 0–26) (Figure 3).

### Clinical Outcome and Disease Severity

Length of hospitalization and PICU admission are summarized in Table 2. Relapse of infection occurred in 3% (25/839). Median time to relapse was 3 days following discharge (IQR, 1–8 days). *Acinetobacter* spp. was the most common organism isolated in those with a relapsed infection (20%, 5/25). No significant difference was observed in the types of comorbidities or presence of CVC in those with relapsed infection (Supplementary section 7).

A total of 48 deaths (5%) were reported, of which 29 (60%) were attributable to GNBSIs. Thirty-day and 90-day in-hospital mortality was 3% and 5%, respectively. Twenty-five percent (12/48) of deaths occurred in neonates and 29% (14/48) in infants. For GNBSI-attributable deaths, median time to death from first positive blood culture was 5 days (IQR, 1–16). Eighty-three percent of deaths occurred in hospital-onset episodes (40/48) and 92% occurred in children with comorbidities (44/48; odds ratio [OR], 4.7; 95% CI, 1.7–13.3 vs previously healthy children). In children who died, cardiovascular, genetic/congenital, neonatal, and respiratory comorbidities were overrepresented (*P* < .05, Supplementary section 8). Pathogen-specific case fatality rates are summarized in Table 4.

**Table 4.** Case Fatality Rates Among Common Gram-negative Pathogens by Their Antimicrobial Resistance Pattern

Organisms	Number of Episodes With Resistant Isolate	CFR From Resistant Pathogen	CFR From Sensitive Pathogen
*E. coli*			
3GC	49/240 (20%)	6/49 (12%)	4/191 (2%)
MDR	115/240 (48%)	7/115 (6%)	3/125 (2%)
*E. cloacae complex*			
3GC	33/90 (37%)	6/33 (18%)	5/57 (9%)
MDR	34/90 (38%)	6/34 (18%)	5/56 (9%)
*P. aeruginosa*			
3GC^a^	20/76 (26%)	2/20 (10%)	2/56 (4%)
MDR	23/76 (30%)	1/23 (4%)	3/53 (6%)
*K. pneumoniae*			
3GC	25/75 (33%)	4/25 (16%)	3/50 (6%)
MDR	27/75 (36%)	4/27 (15%)	3/48 (6%)

Data are n/N (%) and only include episodes with 1 gram-negative organism isolated.

Abbreviations: 3GC, third-generation cephalosporin (ceftazidime and ceftriaxone); CFR, case fatality rate; MDR, multidrug resistant (defined as resistant to ≥3 antimicrobial categories, full details in Supplementary section 2).

^a^Ceftazidime only.

The overall duration of hospitalization was longer in episodes caused by 3GCR-Enterobacterales (17 vs 12 days, *P* < .001) and death occurred more frequently (12% vs 4%, *P* < .001, Table 2). These adverse outcomes were also observed in MDR-Enterobacterales (Supplementary section 2).

Univariable analysis identified age, comorbidities, presence of a CVC, surgery within the past 30 days, ICU requirement (admission to ICU or onset of GNBSIs in the ICU) and infection with 3GCR-Enterobacterales as risk factors for 90-day in-hospital mortality (Table 5). In the final multivariable model, infection with 3GCR-Enterobacterales (OR, 3.2; 95% CI, 1.6–6.4) and ICU requirement (OR, 6.4; 95% CI, 3.0–13.6) were independently associated with death after adjusting for age, comorbidity, and CVC (Table 6).

**Table 5.** Univariate Analysis of Factors Associated With 90-day in-hospital Deaths (n = 44) in Children With GNBSI Across 5 Tertiary Pediatric Hospitals in Australia: 2019–2021

Demographic and Clinical Factors	No. of Deaths/Total	OR	95% CI
Age			
Neonate	12/91 (13%)	4.7	2.2–10.0
Infants 28–364 d	12/197 (6%)	2.0	1.0–4.3
Children aged 1–17 y	20/643 (3%)	(reference)	…
Aboriginal and/or Torres Strait Islander children	4/60 (7%)	1.5	0.5–4.3
Presence of comorbidities	40/661 (6%)	4.3	1.5–12.0
Neutropenia^a^ at time of GNBSI onset	19/309 (6%)	1.6	0.8–2.9
Presence of CVC	38/558 (7%)	4.5	1.9–10.7
Surgery within the past 30 d	20/238 (8%)	2.5	1.4–4.6
ICU admission or onset in ICU	31/205 (15%)	9.8	5.0–19.1
Antimicrobial resistance factor			
Infection with 3GC susceptible Enterobacterales	16/475 (3%)	(reference)	…
Infection with 3GC resistant Enterobacterales	16/129 (12%)	4.1	2.0–8.4
Infection with non-Enterobacterales gram-negative bacteria	12/327 (4%)	1.1	0.5–2.3

Abbreviations: 3GC, third-generation cephalosporin; CI, confidence interval; CVC, central venous catheter; GNBSI, gram-negative bloodstream infection; ICU, intensive care unit; OR, odds ratio.

^a^Defined as neutrophil count <0.5.

**Table 6.** Multivariable Analysis of Factors Associated With 90-day Mortality (n = 44) in Children With GNBSI Across 5 Tertiary Pediatric Hospitals in Australia: 2019–2021

Variables	OR	95% CI
Age		
Neonate	1.9	0.8–4.6
Infants aged 28–364 d	2.0	0.9–4.5
Children aged 1–17 y	(reference)	…
Presence of comorbidities	1.7	0.5–5.7
Presence of CVC	2.5	0.9–6.9
ICU admission or onset of BSI in ICU	6.4	3.0–13.6
Antimicrobial resistance	…	…
Infection with 3GC-susceptible Enterobacterales	(reference)	…
Infection with 3GC-resistant Enterobacterales	3.8	1.7–8.2
Infection with non-Enterobacterales gram-negative bacteria	1.0	0.5–2.3

Abbreviations: 3GC, third-generation cephalosporin; CI, confidence interval; CVC, central venous catheter; GNBSI, gram-negative bloodstream infection; ICU, intensive care unit; OR, odds ratio.

## DISCUSSION

In our large, prospective multicenter study of 931 GNBSIs in hospitalized children, we report that GNBSIs were predominantly community onset, but often healthcare associated. We show that children younger than age 5 years with comorbidities were disproportionately affected. Deaths following GNBSIs occurred more frequently in children with hospital-onset infections and in those with comorbidities. Enterobacterales were the most common pathogens identified and almost a quarter were phenotypically 3GCR, though carriage of known ESBL genes was only confirmed in approximately half. Children with infections caused by 3GCR-Enterobacterales were more likely to have longer hospitalizations and higher rates of mortality.

Australia is a low prevalence country for AMR, especially with regard to carbapenemase-producing Enterobacterales [27]. In our high-income, tertiary children's hospital setting, we observed relatively high rates of AMR, with 22% of Enterobacterales being phenotypically 3GCR. In other high-income settings, large population-based reporting of AMR in invasive childhood infections is limited. Lipworth et al reported a lower rate of 3GC resistance (13%) in UK [4]. A recent systematic review of studies from 5 high-income countries reported that ESBL-producing Enterobacterales contributed to 0%–5% of all pediatric BSIs [28]. The prevalence was higher in neonates with an increase in the proportion of ESBL over time [28]. The multicenter SENTRY surveillance program also reported concerning rates of phenotypic ESBL *Klebsiella* spp. BSI of 18% and 24% in children <1 year and 1–5 years, respectively [29]. We found BSI with 3GCR-Enterobacterales to be independently associated with increased risk for death. 3GCs are commonly the first-line empirical antibiotics for pediatric sepsis; therefore with an increasing burden of 3GCR-Enterobacterales, prompt identification of the causative organism and associated AMR may help to optimize targeted antibiotic selection.

Through WGS, we identified substantial intraspecies diversity and variable carriage of AMR genes with significant differences in STs observed between sites. The dominant *E. coli* ST we observed was similar to those previously reported [2, 4, 30]. However, of particular concern is the emergence of ST1193 in Australian children, which has been described as a high-risk MDR clone [31]. It has been increasingly described in neonates in China and USA [32, 33].

It is a common presumption that 3GC resistance in Enterobacterales is secondary to ESBL production. Mechanisms such as AmpC are less well recognized. Whole genome sequencing allowed us to characterize 3GCR-Enterobacterales that were not ESBL. The most common plasmid-mediated AmpC genes seen in our study were bla*_DHA1_* and *bla*_CMY_, which is consistent with what is reported globally [34]. An understanding of non-ESBL mechanisms of 3GC resistance in Enterobacterales is important because alternatives to carbapenem therapy may be possible. Although carbapenem resistance for Enterobacterales was infrequent in this study, it was observed in 16% and 5% of *A. baumannii* and *P. aeruginosa,* respectively. This will need further attention because optimal treatment of these resistant gram-negative infections in children is unclear, with limited evidence for newly developed antimicrobials in children.

Case fatality following sepsis in children in high-income settings has been reported to be up to 7% [1]. We observed a lower overall mortality of 3%. Similar to a Swiss study of culture-proven sepsis, we also saw a higher case fatality in neonates and children with comorbidities [1]. Poorer outcomes following resistant gram-negative infections in children have been described but limited to small case series and in those with specific conditions such as malignancies [7, 35]. Here, we demonstrated this across a large and diverse group of children.

Our study has considerable strengths. We report 931 clinically significant episodes of GNBSIs in children across Australia, accompanied by detailed clinical, microbiological, and molecular characterization. Our findings address an important knowledge gap because most previous studies of GNBSIs in children are either limited to specific clinical conditions or limited in sample size, reducing the generalizability of the findings.

There are limitations to our study. Our inclusion of patients from tertiary children's hospitals may not be representative of previously healthy children treated in other settings. Despite the relatively large number of GNBSI episodes captured, the number of deaths were small, which limited our exploration of risk factors. Importantly, because antimicrobial therapy was highly variable, we were cautious in drawing associations with clinical outcomes, though we acknowledge that early exposure to effective antimicrobial therapy is likely to improve outcomes. A detailed analysis of the relationship between antimicrobial therapy and clinical outcomes will form the basis of future work. Finally, there were a few carbapenem-resistant Enterobacterales for which no known carbapenemase genes were identified. This is likely due to plasmid loss during subculture.

Invasive gram-negative infections are an increasing problem in neonates and children, further compounded by worsening AMR and limited access to effective antibiotics [11, 36]. There is a global shortage of antibiotic clinical trials in neonates and children [10], and antibiotic development programs rarely include these vulnerable groups. It is an urgent priority that neonates and children are included in future antibiotic clinical trials.

## CONCLUSION

Children hospitalized with GNBSIs were young and often medically complex. GNBSIs caused by 3GCR-Enterobacterales were associated with death. These findings should inform the design of trials to determine the optimal management of GNBSIs in children.

## Supplementary Data

Supplementary materials are available at *Clinical Infectious Diseases* online. Consisting of data provided by the authors to benefit the reader, the posted materials are not copyedited and are the sole responsibility of the authors, so questions or comments should be addressed to the corresponding author.

## Supplementary Material

ciae341_Supplementary_Data

## Notes

***Acknowledgments.*** PAEDS network Group in addition to named authors: Jeremy Carr, Nan Vasilunas, Joshua R. Francis, Kristine Macartney, Nigel W. Crawford, Nicholas Wood, Jim Buttery, Helen Marshall, Elizabeth Elliott, Peter Richmond, Emma Carey, Alissa McMinn, Karen Bellamy, Guillian Hunter, Kathryn Meredith, Laura Rost, Nicole Kerly, Sara Cook, Natasha Doran, Laura Francis, Christine Heath and Carolyn Finucane, Mehyar Khair Baik, Laura Lopez, and Catherine Glover.

The authors also acknowledge Dominika Butkiewicz, Chelsie Smith, Alison Kesson, Alex Outhred, Fungai Mukungunurwa, Jacinta Bowman, Avram Levy, and scientists from the bacteriology laboratory at Children's Hospital Westmead for their laboratory assistance and expertise.

***Financial support*.** This work was supported by Queensland Health Advancing Clinical Research Fellowship to S. C. H. W. A. D. I. was supported by National Health and Medical Research Council Grant (NHMRC, grant ID 1197743) and Children's Hospital Foundation Accelerator Grant (grant number 50314). C. L. L. was supported by an National Health and Medical Research Council (NHMRC) Investigator Grant (1193826). G. S. was supported by an National Health and Medical Research Council (NHMRC) Emerging Leader Fellowship (APP1173857). P. N. B. was supported by an National Health and Medical Research Council (NHMRC) Early Career Fellowship (APP1145817).
