# Supplementary material for: Characterization of Gram-negative Bloodstream Infections in Hospitalized Australian Children and Their Clinical Outcomes
Source: Clin Infect Dis. 2024 Jun 25;79(3):734–43. doi: 10.1093/cid/ciae341 (PMC11426278; doi:10.1093/cid/ciae341)

**Supplementary materials**

**Section 1 Definition of bloodstream infection**

Bloodstream infection is defined as per the Australian Commission on Safety and Quality in Healthcare, which must meet the conditions in one of the following criteria:

Criterion 1 (recognised pathogens; non neonatal intensive care)

Isolation of one or more recognised bacterial or fungal pathogens from one or more blood cultures (e.g. *Staphylococcus aureus*, *Streptococcus pneumoniae*, *Escherichia coli*, *Klebsiella*, *Proteus*, *Salmonella species*, *Candida albicans*).

Note: Where mixed isolates are obtained with one being an accepted pathogen, the potential contaminant* organism is to be disregarded.

Criterion 2 (potential contaminants* in patients aged >1 year)

The patient has at least one of the following signs and symptoms within 24 hours of a positive blood culture being collected:

Fever (>38°C); Chills or rigors; or Hypotension

and

at least one of the following:

1. there is isolation of the same potential contaminant* from two (2) or more blood cultures drawn on separate occasions within a 48 hour period (isolates identified by suitable microbiological techniques)
2. there is isolation of a potential contaminant* from a single blood culture drawn from a patient with an intravascular line (within 48 hours of the episode) and appropriate antimicrobial therapy against that isolate is commenced.

Criterion 3 (potential contaminants* in patients aged <1 year)

The patient has at least one of the following signs and symptoms within 24 hours of a positive blood culture being collected:

fever (>38°C); hypothermia (<36°C); or apnoea or bradycardia

and

at least one of the following:

1. there is isolation of a potential contaminant* from two (2) or more blood cultures drawn on separate occasions within a 48 hour period.
2. there is isolation of a potential contaminant* from a single blood culture drawn from a patient with an intravascular line (within 48 hours of the episode) and appropriate antimicrobial therapy is commenced.

*Potential contaminant organisms include coryneforms (Corynebacterium, etc.), coagulase-negative staphylococci, micrococci, Propionibacterium, Bacillus, alpha haemolytic streptococci, environmental Gram-negative bacilli, non-pathogenic Neisseria.

**Section 2 MDR vs non-MDR Enterobacterales**

MDR is defined as resistance against ≥1 agent in ≥3 antimicrobial categories.

Antimicrobials included in the analysis were:

Aminoglycosides (gentamicin, tobramycin, amikacin), antipsuedomonal penicillins + beta-lactamse inhibitors (piperacillin-tazobactam), carbapenems (ertapenem, imipenem, meropenem, doripenem), extended-spectrum cephalosporins (ceftriaxone, ceftazidime, cefepime), fluoroquinonlones (ciprofloxacin), folate pathway inhibitors (trimethoprim-sulphamethoxazole), monobactams (aztreonam), penicillins (ampicillin), penicillins + beta-lactamase inhibitors (ampicillin-sulbactam) and polymyxins (colistin).

| # of antimicrobial resistant categories | N (%) |
| --- | --- |
| 0 | 256 (39%) |
| 1 | 110 (17%) |
| 2 | 70 (11%) |
| 3 | 68 (10%) |
| 4 | 64 (10%) |
| 5 | 35 (5%) |
| 6 | 22 (3%) |
| 7 | 24 (4%) |
| 8 | 7 (1%) |
| 9 | 3 (0.5%) |
| Total | 659 |

|  | Total N=931 | MDR N=219 | Non-MDR N=405 | P value |
| --- | --- | --- | --- | --- |
| PICU admission required for GNBSI onset out of PICU n (%) | 100 (11%) | 27 (12%) | 36 (9%) | 0.17 |
| GNBSI onset in PICU  n (%) | 105 (11%) | 27(12%) | 45 (11%) | 0.65 |
| Invasive ventilation support n (%) | 103 (11%) | 28 (13%) | 40 (10%) | 0.28 |
| Inotropic support n (%) | 88 (9%) | 23 (11%) | 40 (10%) | 0.8 |
| Renal replacement therapy support n (%) | 9 (1%) | 3(1%) | 4 (1%) | 0.70 |
| Relapse within 14 days n (%) | 25 (3%) | 3 (1%) | 10 (2%) | 0.35 |
| Duration of hospitalisation (median days, IQR) | 12 (7-24) | 15 (10-29) | 11 (7-21) | <0.001 |
| Duration of PICU admission overall†  (median days, IQR) | 8 (2-25) | 5.5 (2-28) | 8 (3-19) | 0.72 |
| Duration of PICU admission for episodes onset out of PICU  (median days, IQR) | 3 (1-7) | 2 (2-7) | 3 (2-8) | 0.62 |
| All causes of deaths  n (%) | 48 (5%) | 20 (9%) | 15 (4%) | 0.005 |
| GNBSI related deaths  n (%) | 29 (3%) | 7 (2%) | 13 (6%) | 0.004 |
| 30-day in-hospital mortality  n (%) | 32 (3%) | 14 (6%) | 8 (2%) | 0.004 |
| 90-day in-hospital mortality n (%) | 44 (5%) | 19 (9%) | 13 (3%) | 0.003 |

†Includes admissions that were not initially due to GNBSI

GNBSI = Gram-negative bloodstream infection

PICU = Paediatric intensive care unit

**Section 3**

**Site-specific incidences**

| Incidence | 2019 | 2020 | 2021 |
| --- | --- | --- | --- |
| Overall* | 0.00209 | 0.002 | 0.00184 |
| Overall per 100,000 hospitalisation* | 210 | 210 | 180 |
| Site 1 (per 1000 hosp) | 190 | 200 | 170 |
| Site 2 (per 1000 hosp) | N/A | 240 | 130 |
| Site 3 (per 1000 hosp) | 200 | 200 | 210 |
| Site 4 (per 1000 hosp) | 330 | 280 | 330 |
| Site 5 (per 1000 hosp) | 130 | 100 | 70 |
| *Site 2 excluded in 2019 calculations | |  |  |

**BSI onset by site**

|  | **Total** | **Site 5** | **Site 3** | **Site 4** | **Site 2** | **Site 1** | **p-value** |
| --- | --- | --- | --- | --- | --- | --- | --- |
|  | **N=931** | **N=90** | **N=249** | **N=300** | **N=71** | **N=221** |  |
| BSI onset |  |  |  |  |  |  | <0.001 |
| Community | 579 (62.2%) | 75 (83.3%) | 159 (63.9%) | 147 (49.0%) | 51 (71.8%) | 147 (66.5%) |  |
| Hospital | 352 (37.8%) | 15 (16.7%) | 90 (36.1%) | 153 (51.0%) | 20 (28.2%) | 74 (33.5%) |  |

BSI = Bloodstream infection

**Section 4 Microbiology by presence of comorbidities**

| Organism | Total N=931 | No comorbidities N=269 | Comorbidities present N=662 | P value |
| --- | --- | --- | --- | --- |
| *E. coli* | 251 | 96 (36%) | 155 (23%) | <0.001 |
| *K. pneumoniae* | 97 | 4 (1%) | 93 (14%) | <0.001 |
| *E. cloacae complex* | 108 (12%) | 5 (2%) | 103 (16%) | <0.001 |
| *S. marcescens* | 14 (2%) | 0 (0%) | 14 (2%) | 0.016 |
| *P. aeruginosa* | 85 (9%) | 4 (1%) | 81 (12%) | <0.001 |
| *Salmonella spp* | 97 (10%) | 87 (32%) | 10 (2%) | <0.001 |
| *S. maltophilia* | 23 (2%) | 2 (1%) | 21 (3%) | 0.030 |
| *A. baumannii* | 14 (2%) | 2 (1%) | 12 (2%) | 0.22 |

**Section 5**

***K.pneumoniae* ST and site**


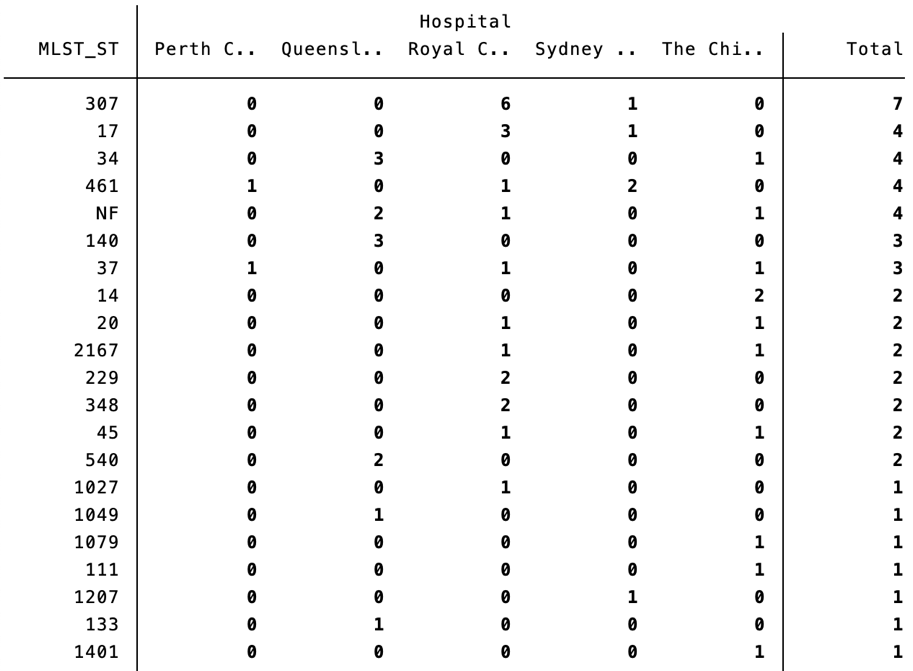

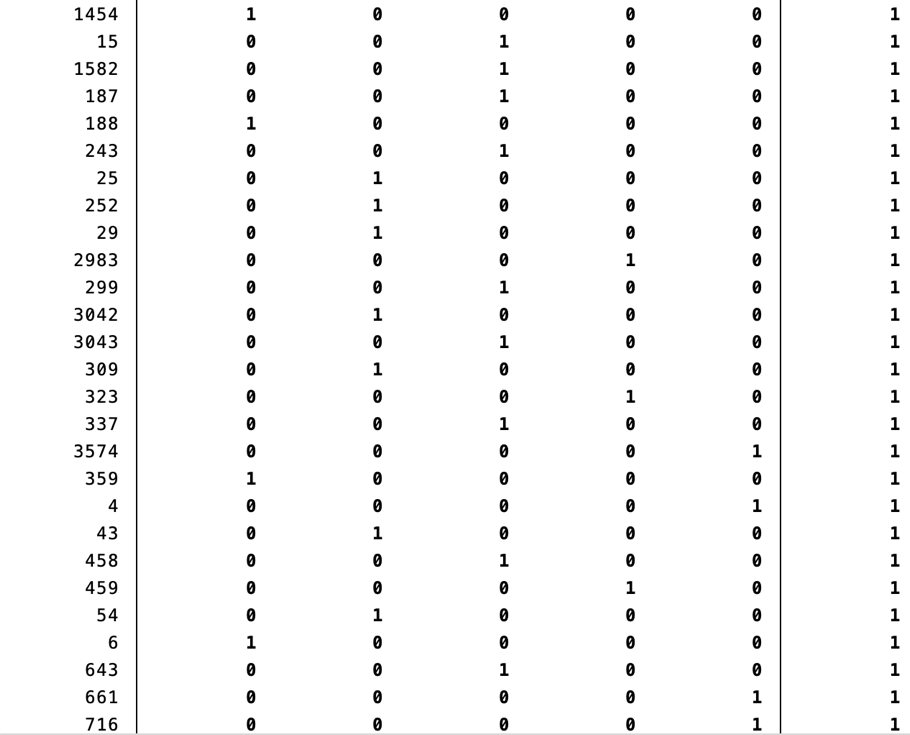


Site 1

Site 2

Site 4

Site 3

Site 5


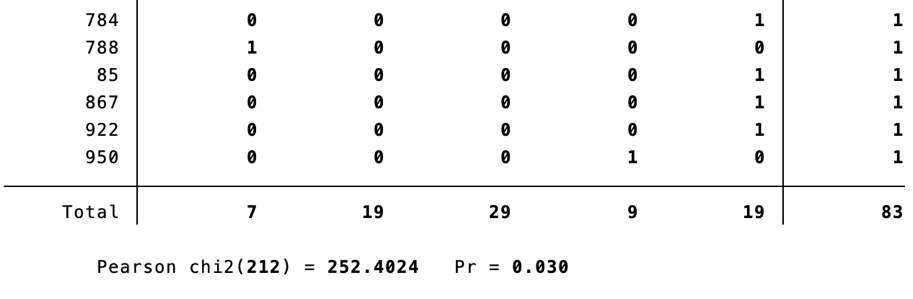


***S. enterica* ST and site**

**
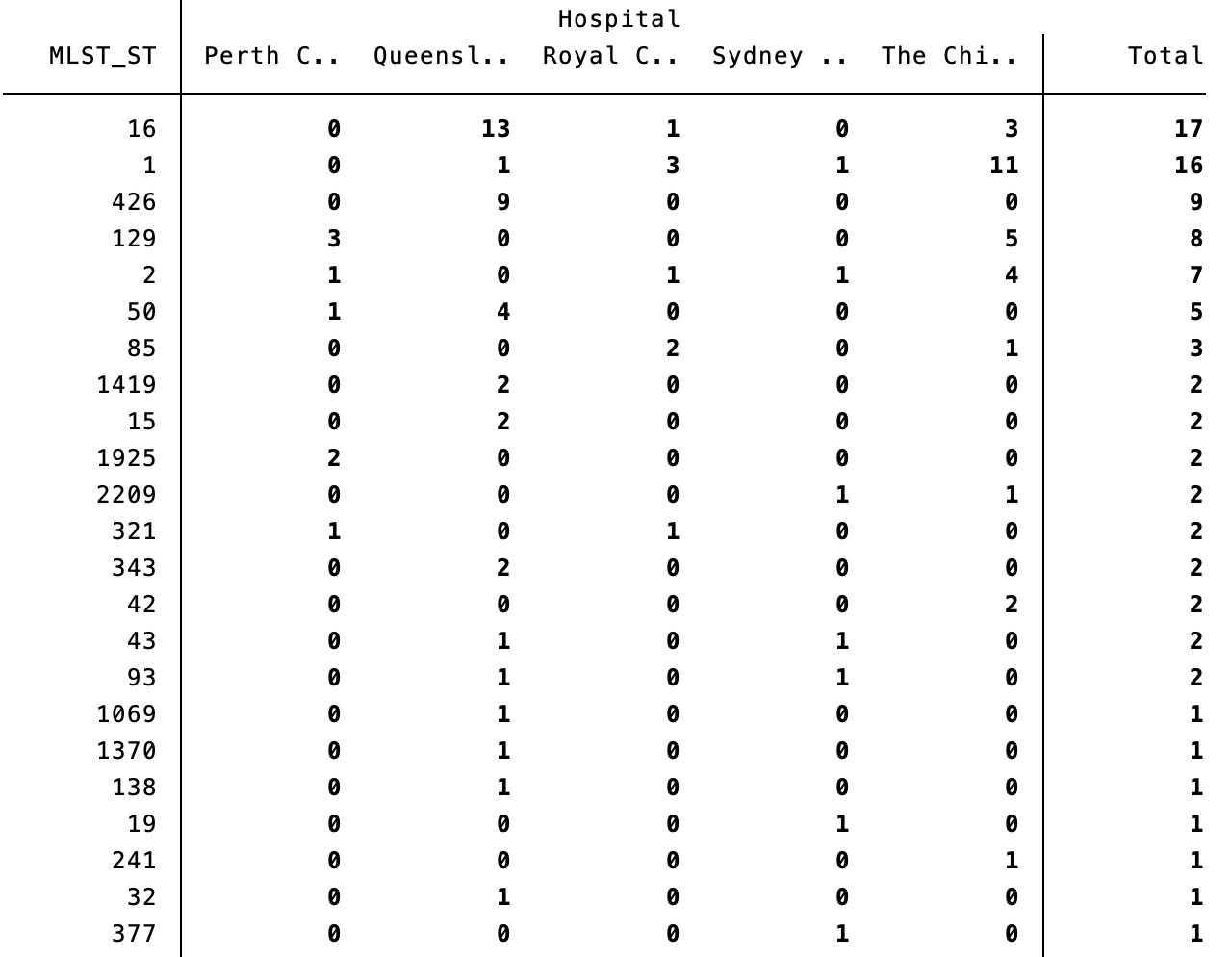

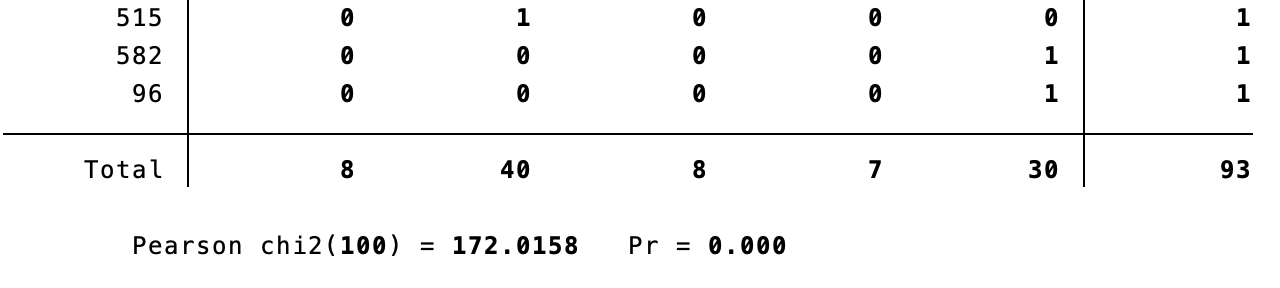
**

Site 1

Site 2

Site 4

Site 3

Site 5

***P. aeruginosa* ST and site**

Site 4

**
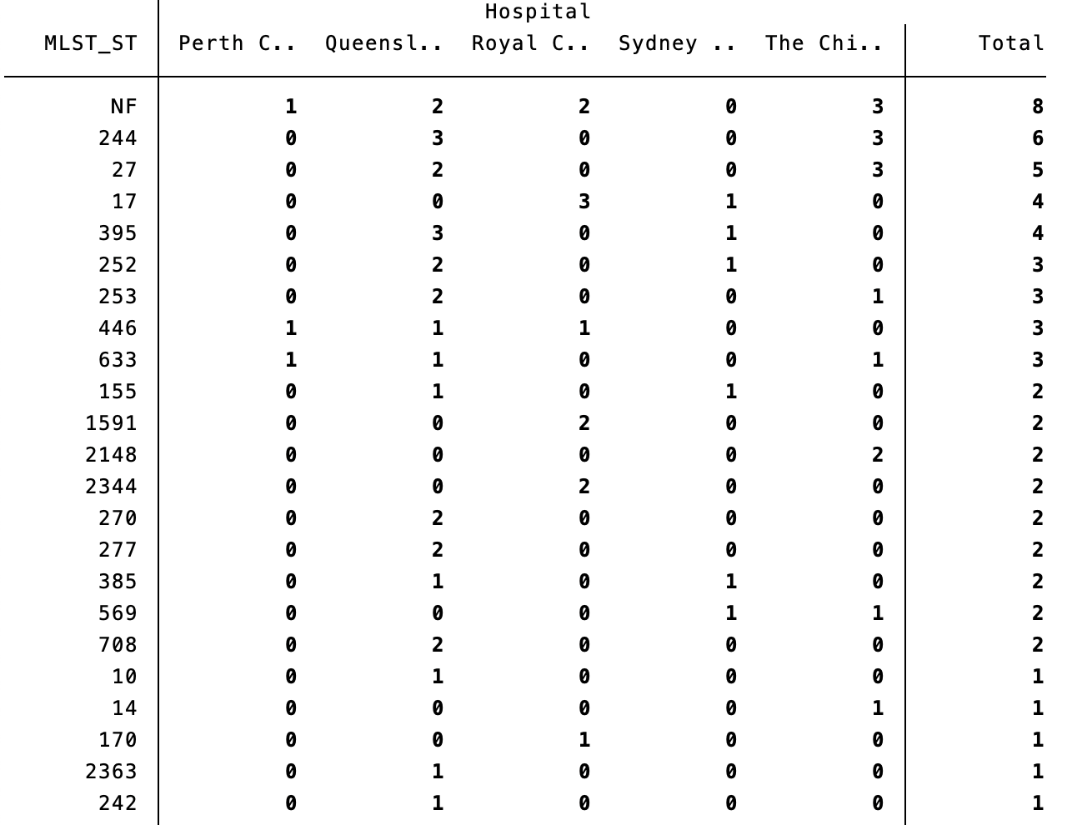

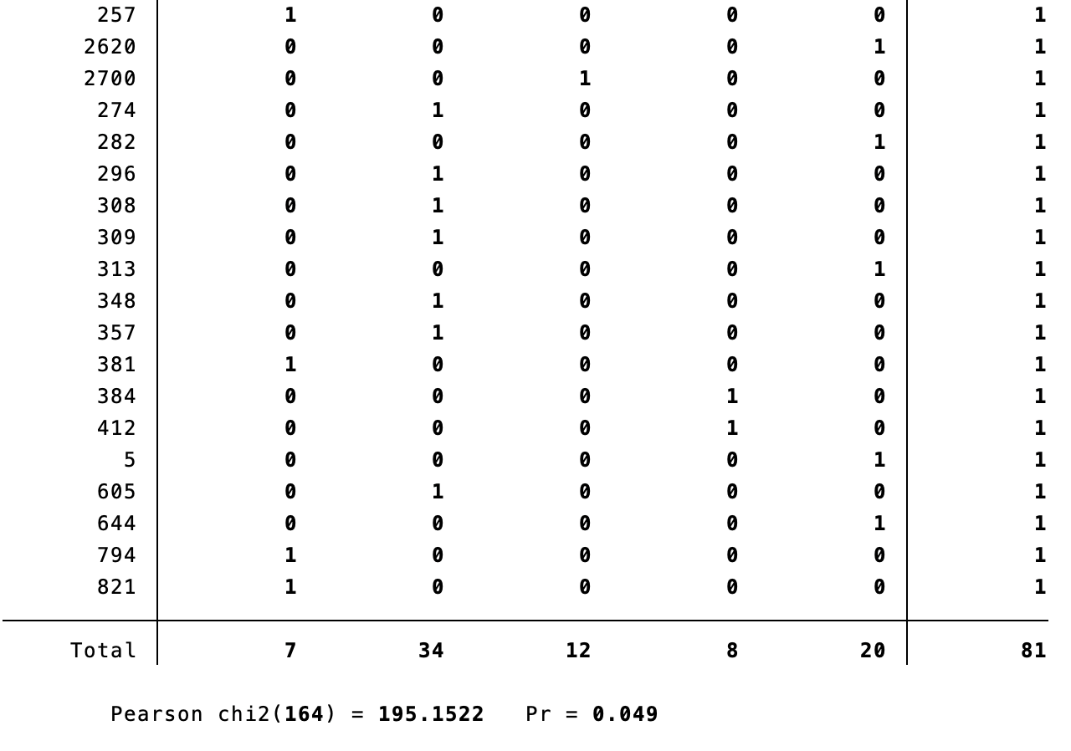
**

Site 1

Site 2

Site 3

Site 5

***E. hormaechei* ST and sites**

Site 3

Site 5

**
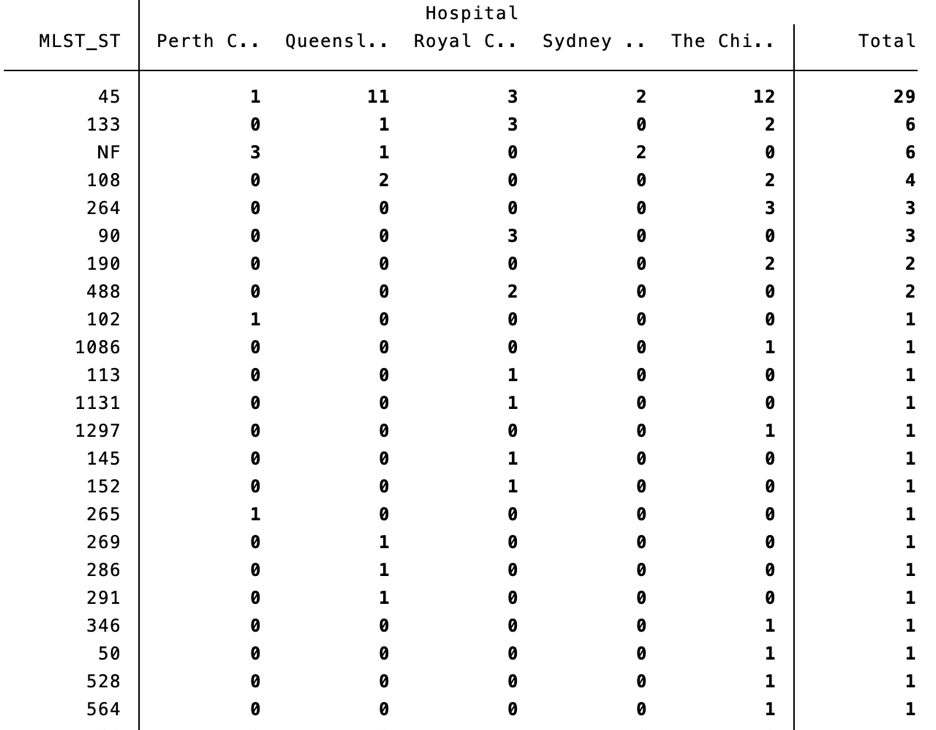

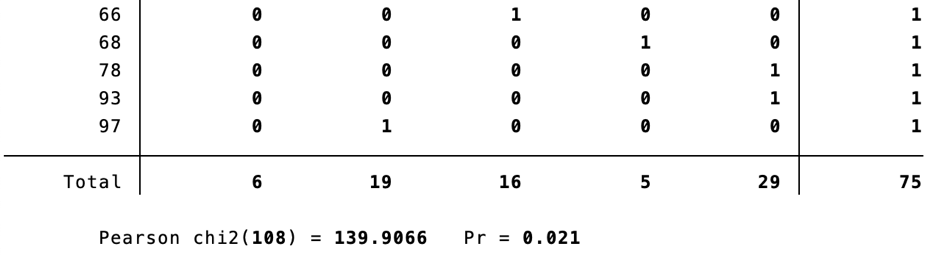
**

Site 1

Site 2

Site 4

***E.coli* ST and age group**


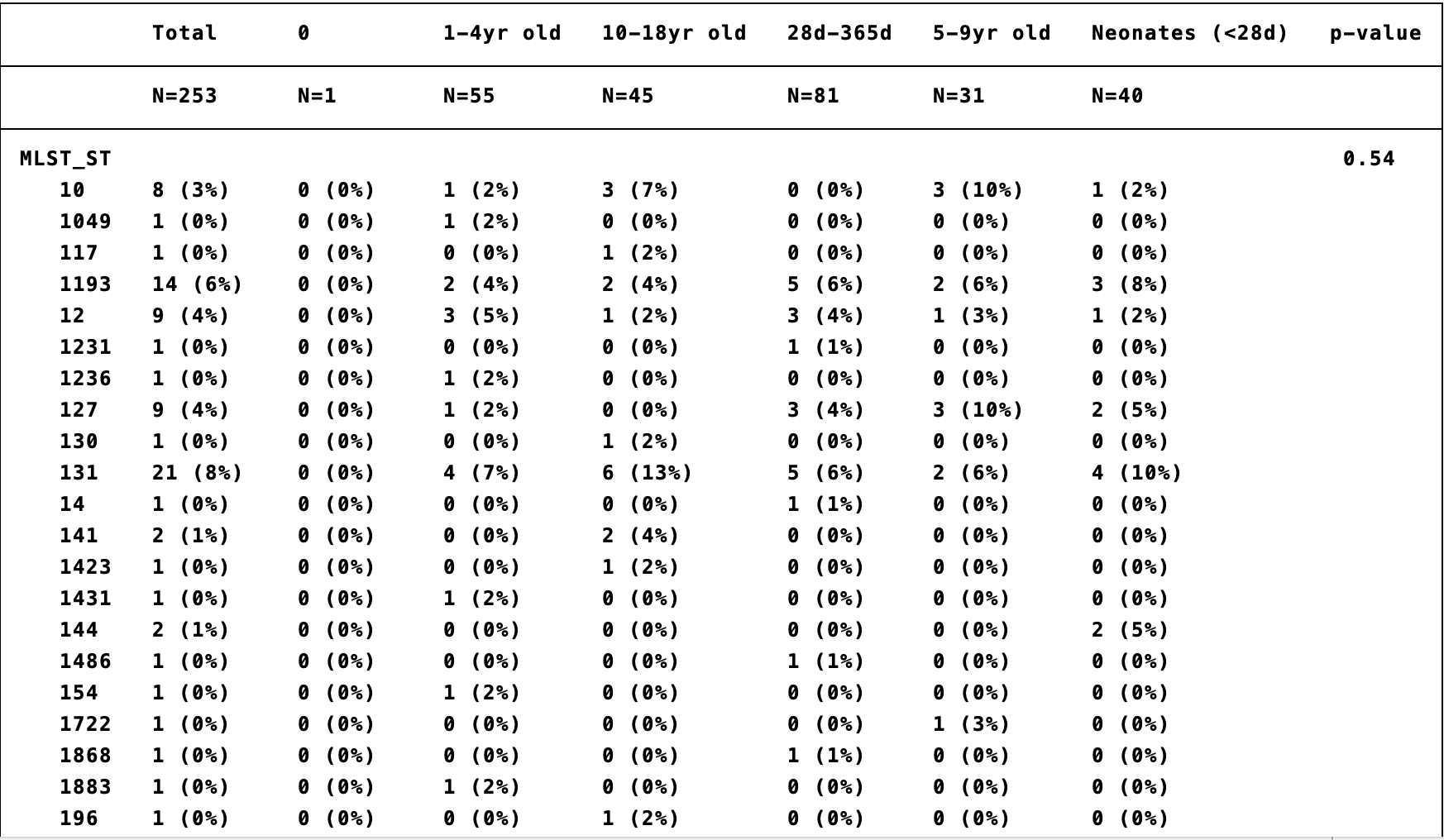

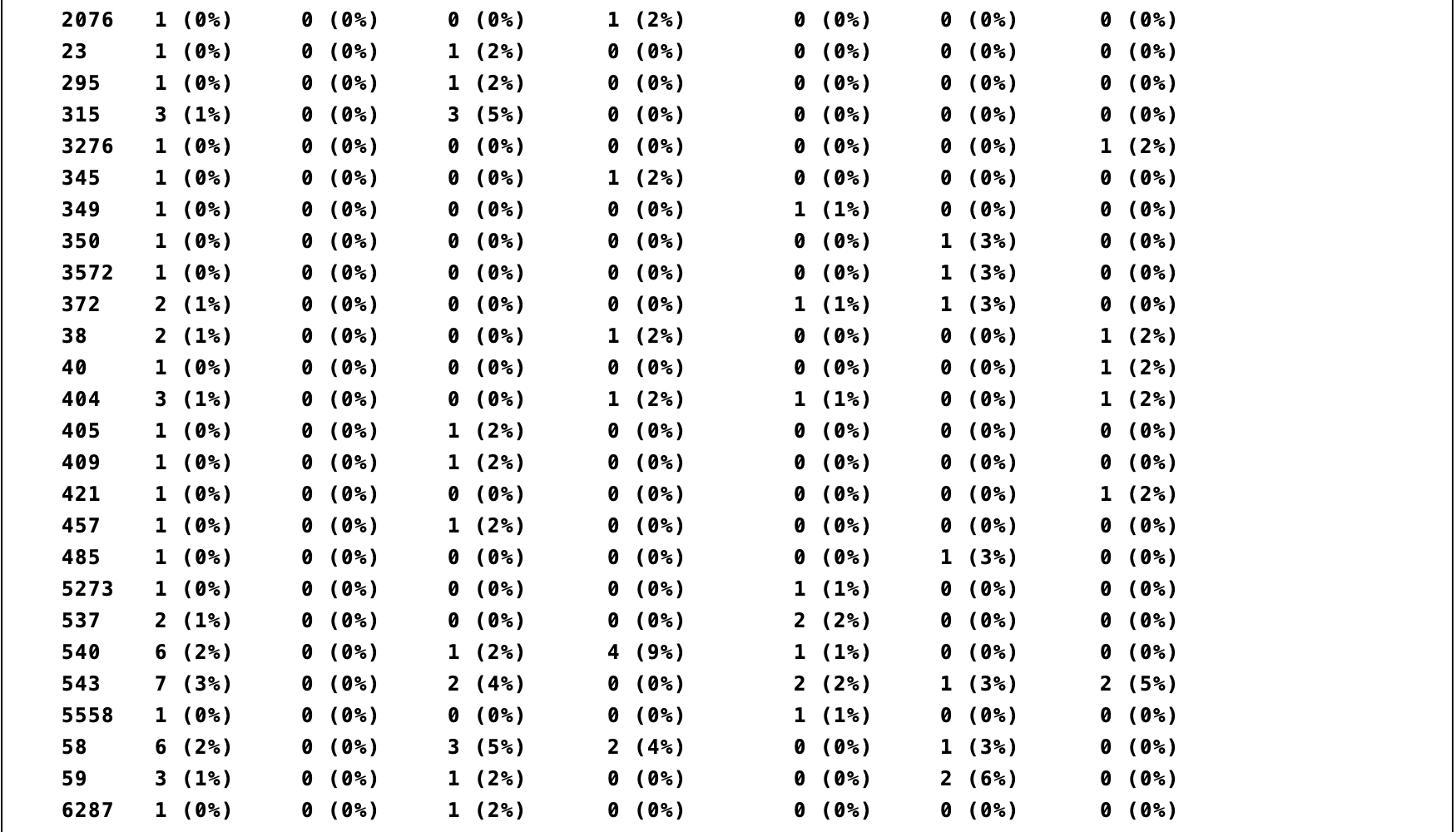

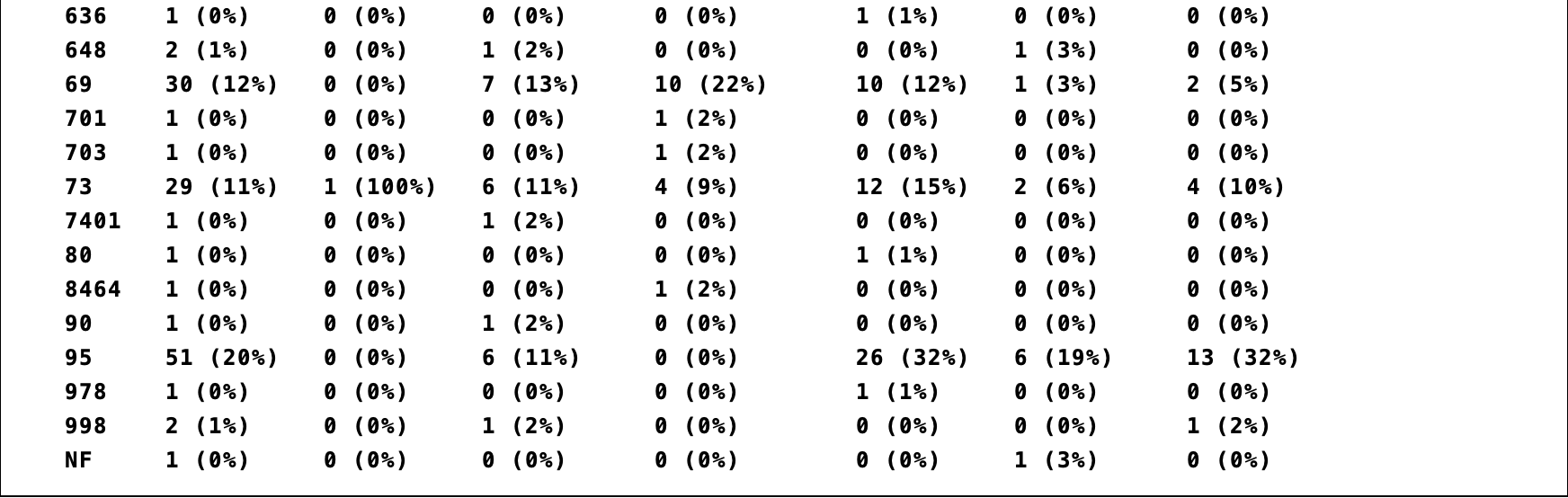

***E.coli ST* and comorbidity**


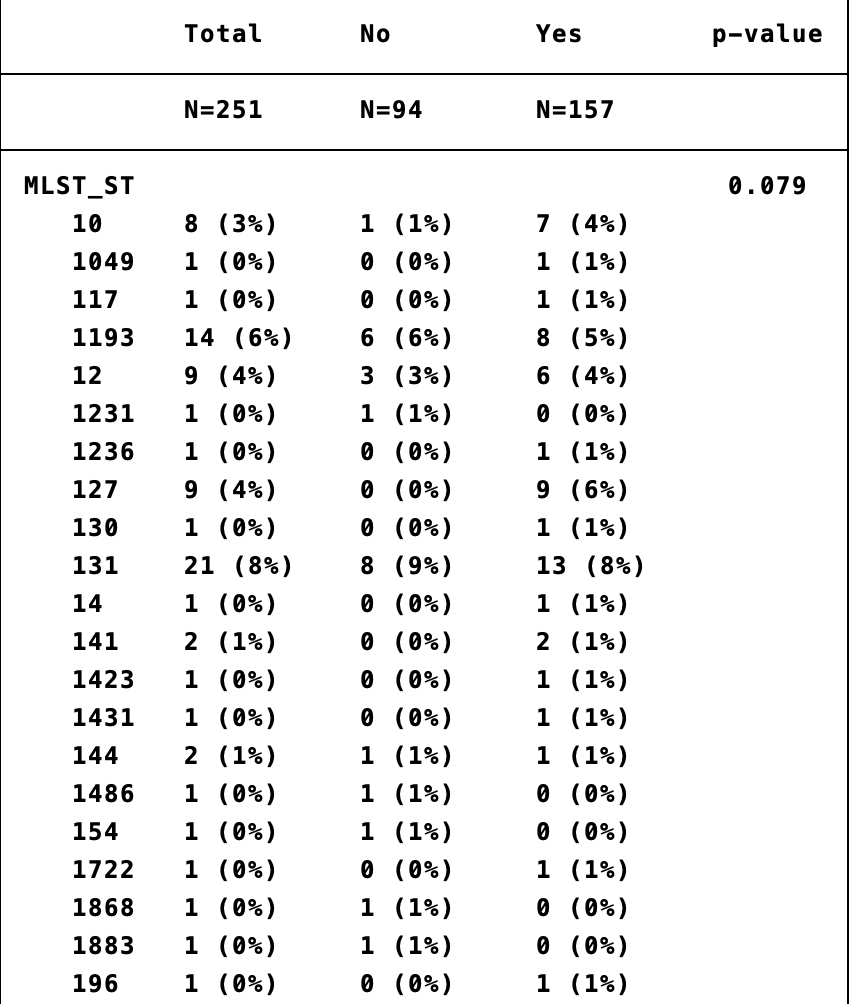


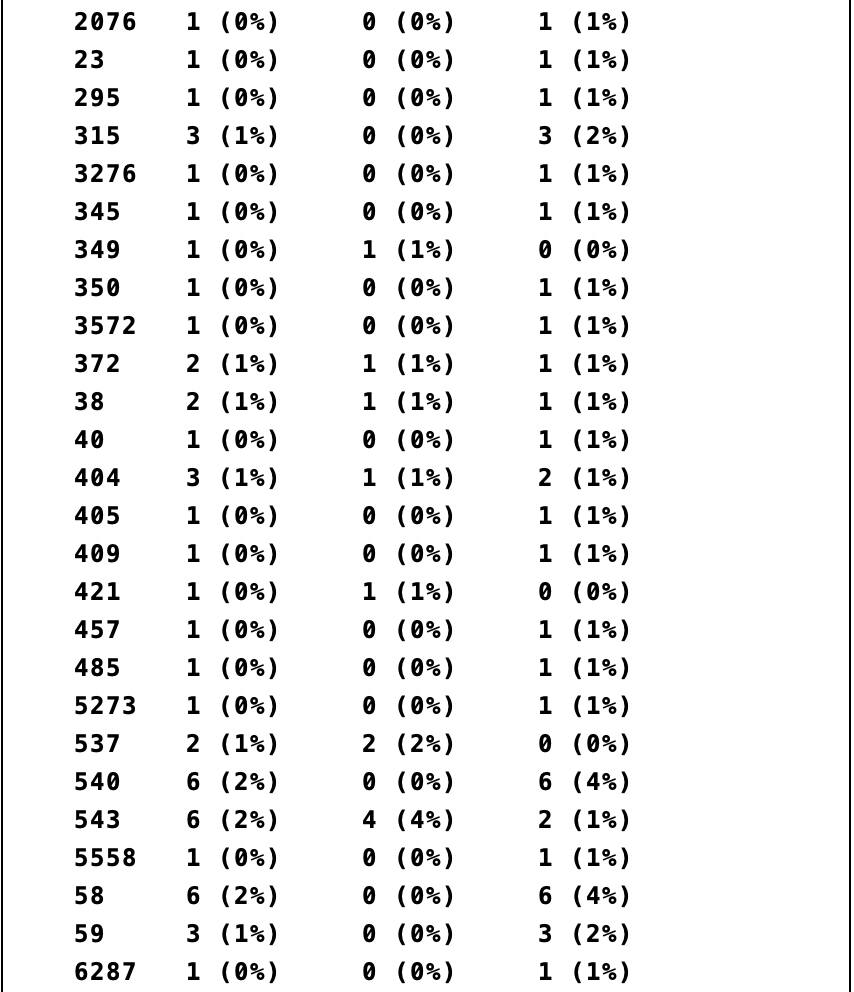


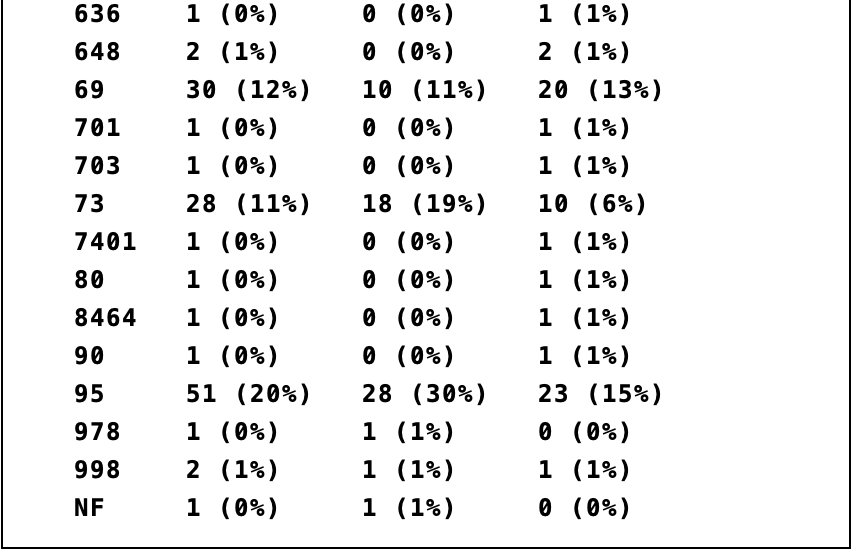


***E.coli ST* and clinical outcome**


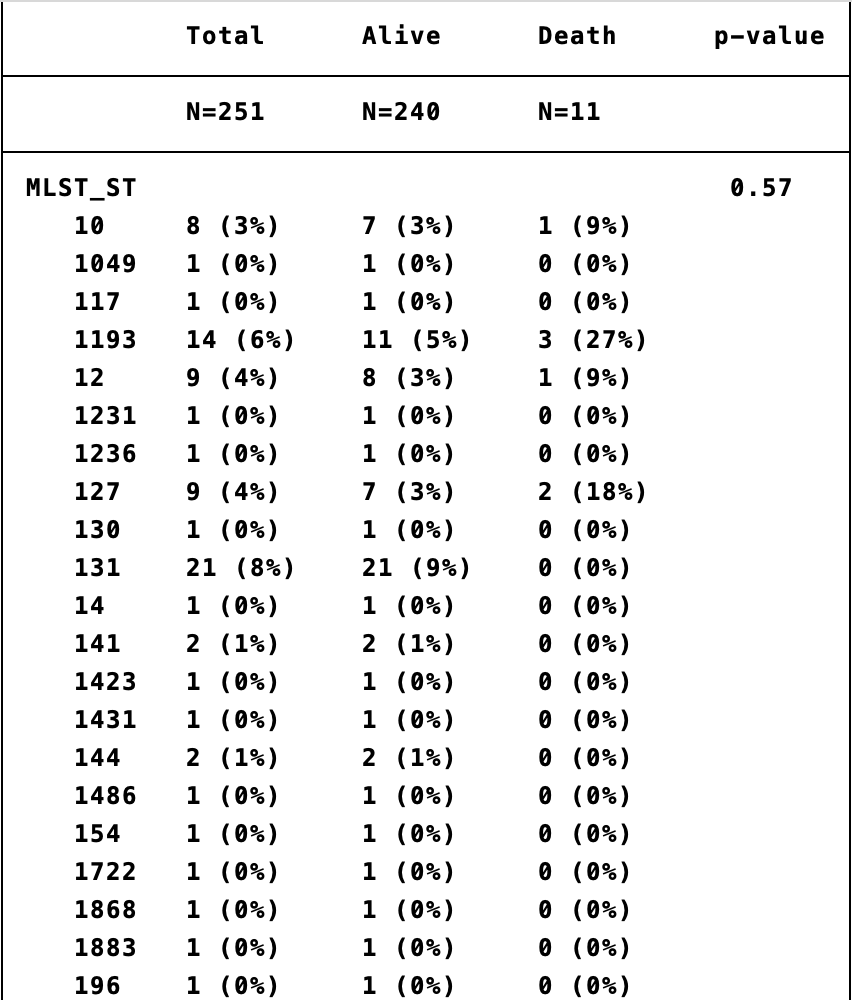

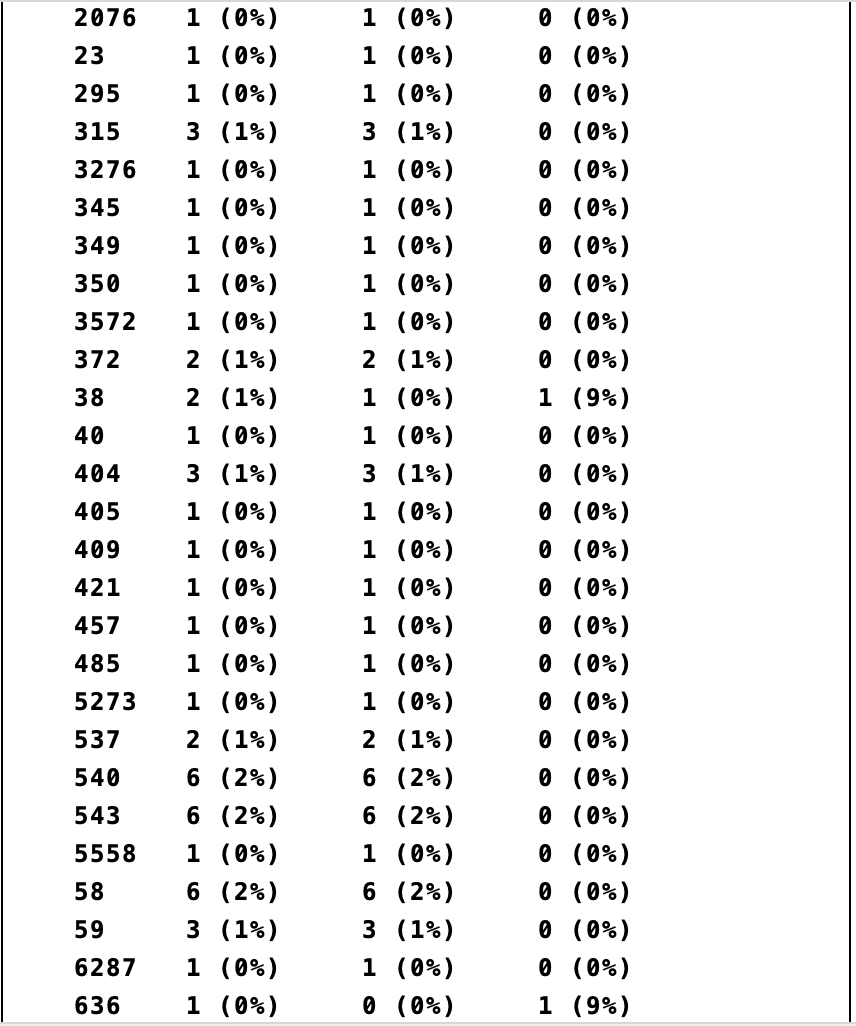

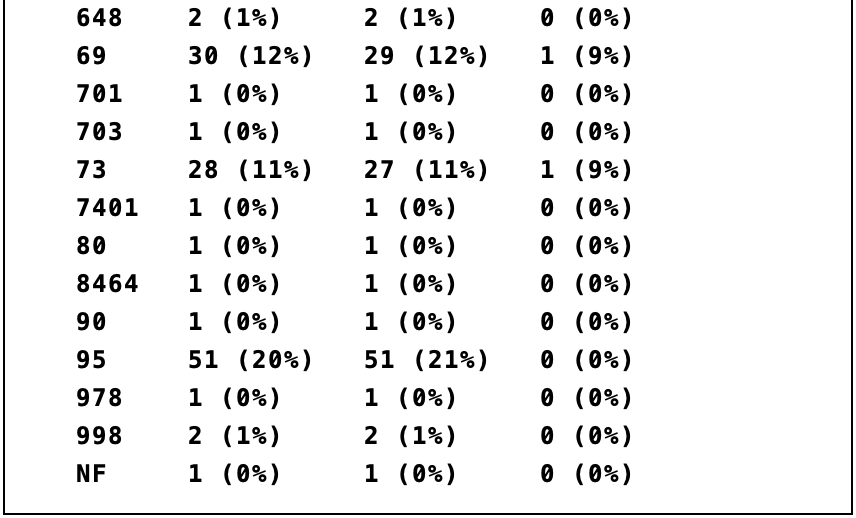


***E.coli ST* and focus of infection**


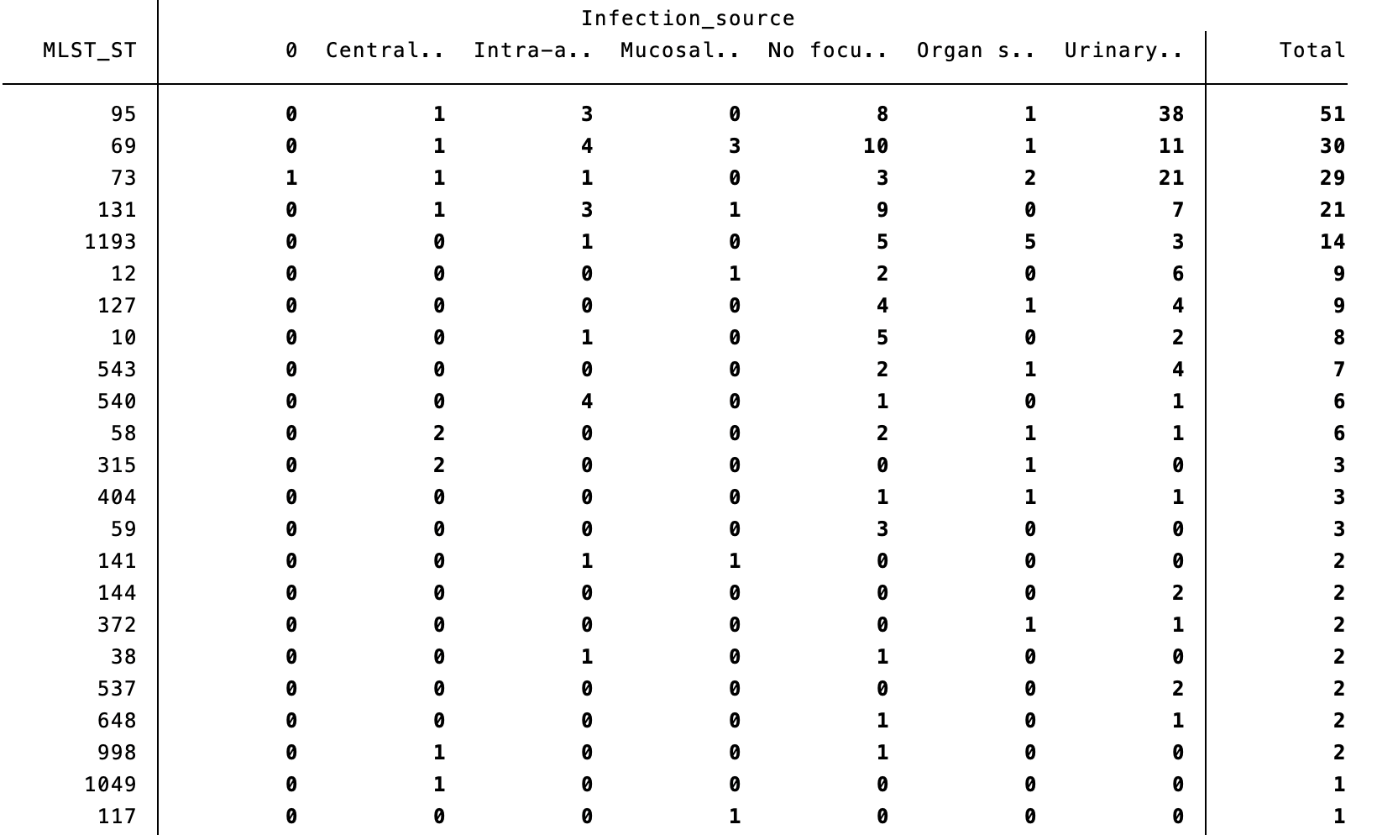

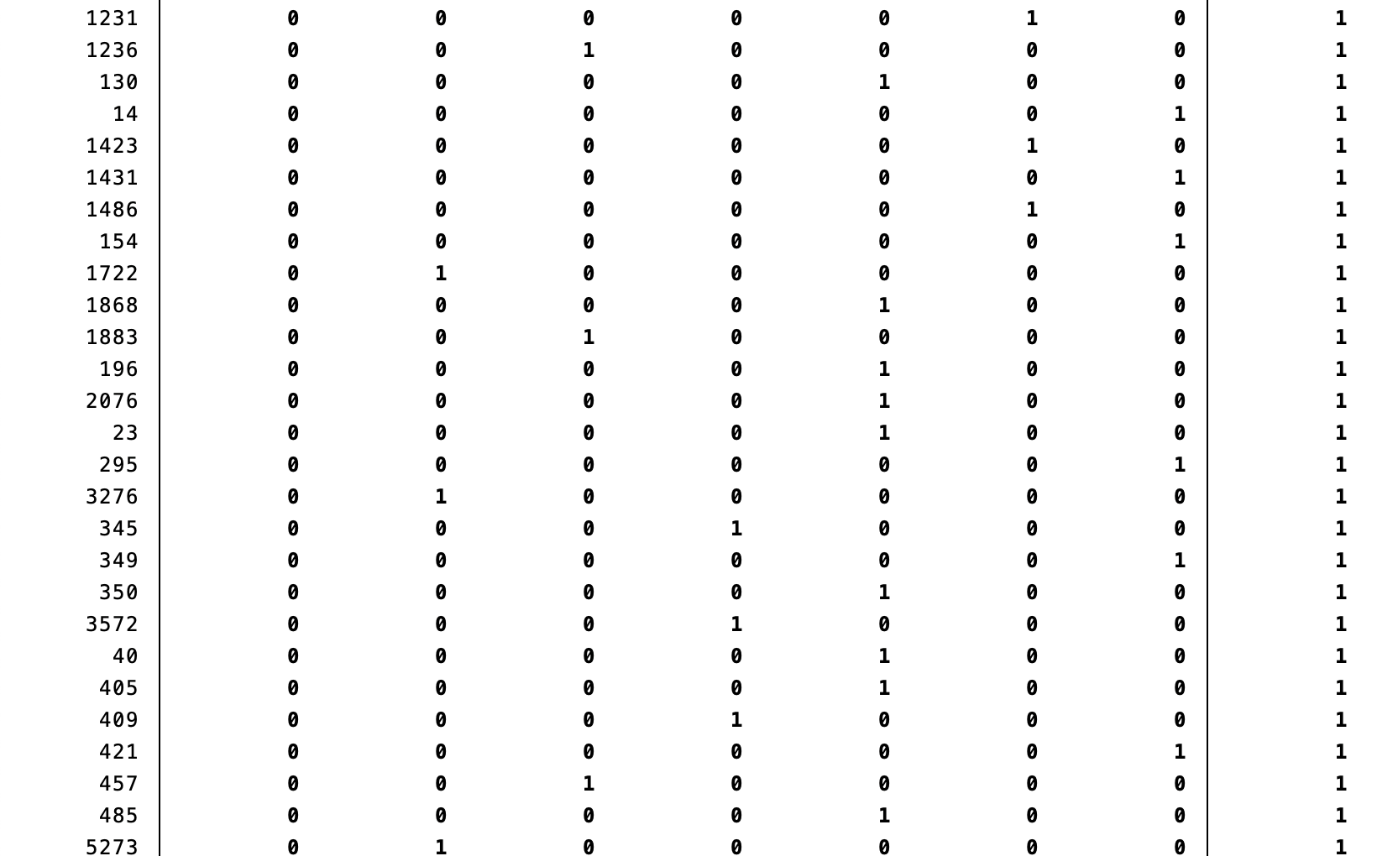

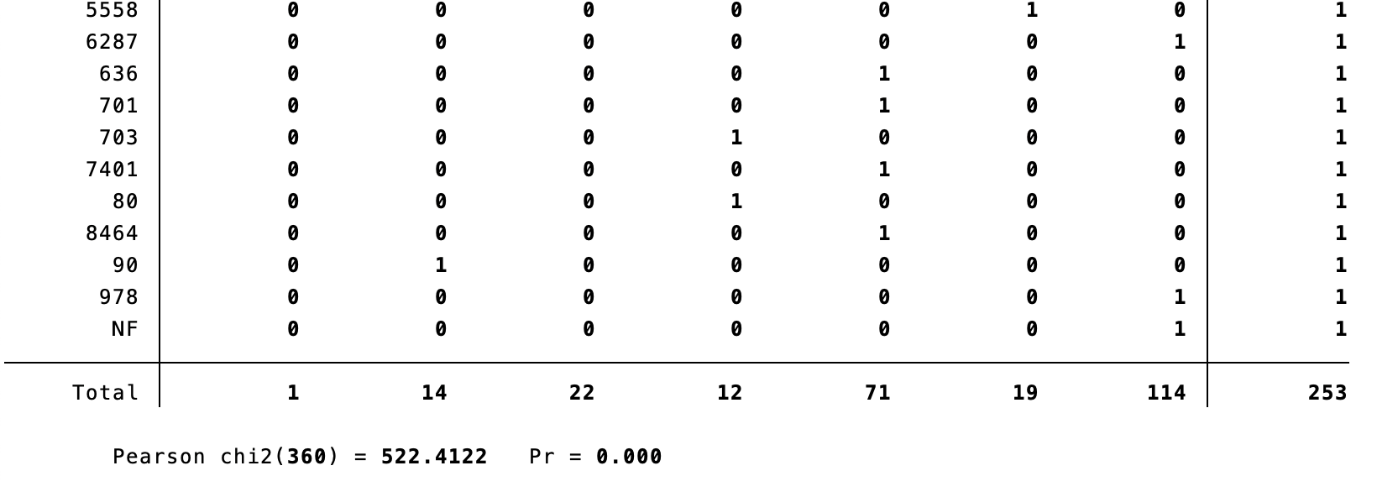

**Section 6 Aminoglycoside resistance rates**

|  |  | % resistant | | |
| --- | --- | --- | --- | --- |
| Organism | Aminoglycoside | Gentamicin | Amikacin | Tobramycin |
| Enterobacterales (n=659)* | 19% | 13% | 3% | 15% |
| *E. coli* (n=256) | 23% | 16% | 3% | 18% |
| *E. cloacae complex* (n=109) | 22% | 16% | 3% | 19% |
| *K. pneumoniae* (n=97) | 26% | 16% | 3% | 21% |
| *S. marcescens* (n=15) | 43% | 14% | 14% | 36%* |
| *P. aeruginosa* (n=81) | 16% | N/A | 7% | 14% |
| *A. baumannii* (n=14) | 29% | 14% | 7% | 29% |

*Serratia species may contain chromosomal genes that mediate intrinsic low-level resistance to tobramycin

**Section 7**

**Readmission by presence of indwelling CVC (1 = readmission)**


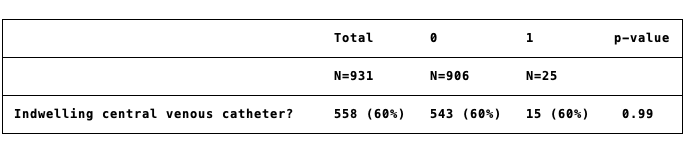


**Readmission by types of comorbidities (1 = readmission)**


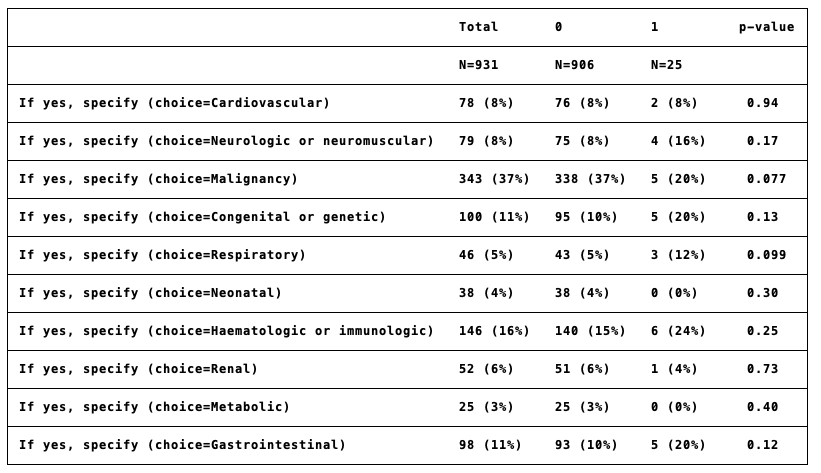


**Section 8 Comorbidities and death (1 = death)**

|  | **Total** | **0** | **1** | **p-value** |
| --- | --- | --- | --- | --- |
|  | **N=931** | **N=883** | **N=48** |  |
| Cardiovascular | 78 (8%) | 65 (7%) | 13 (27%) | <0.001 |
| Neurologic or neuromuscular | 79 (8%) | 73 (8%) | 6 (12%) | 0.31 |
| Malignancy | 343 (37%) | 328 (37%) | 15 (31%) | 0.41 |
| Congenital or genetic | 100 (11%) | 88 (10%) | 12 (25%) | 0.001 |
| Respiratory | 46 (5%) | 39 (4%) | 7 (15%) | 0.002 |
| Neonatal | 38 (4%) | 33 (4%) | 5 (10%) | 0.023 |
| Haematologic or immunologic | 146 (16%) | 136 (15%) | 10 (21%) | 0.31 |
| Renal | 52 (6%) | 49 (6%) | 3 (6%) | 0.84 |
| Metabolic | 25 (3%) | 21 (2%) | 4 (8%) | 0.013 |
| Gastrointestinal | 98 (11%) | 89 (10%) | 9 (19%) | 0.057 |

**Section 9 Surgical procedures undertaken within 30 days of GNBSI**

| **Type of surgery (N=300)** | **n (%)** |
| --- | --- |
| Cardiac | 27 (11%) |
| Ear, nose, throat | 6 (3%) |
| Gastrointestinal | 42 (18%) |
| Genitourinary | 7 (3%) |
| Neurosurgical | 5 (2%) |
| Orthopaedic | 9 (4%) |
| Other | 142 (60%) |

**Section 10 Antimicrobial therapy data in *E. coli* BSI**


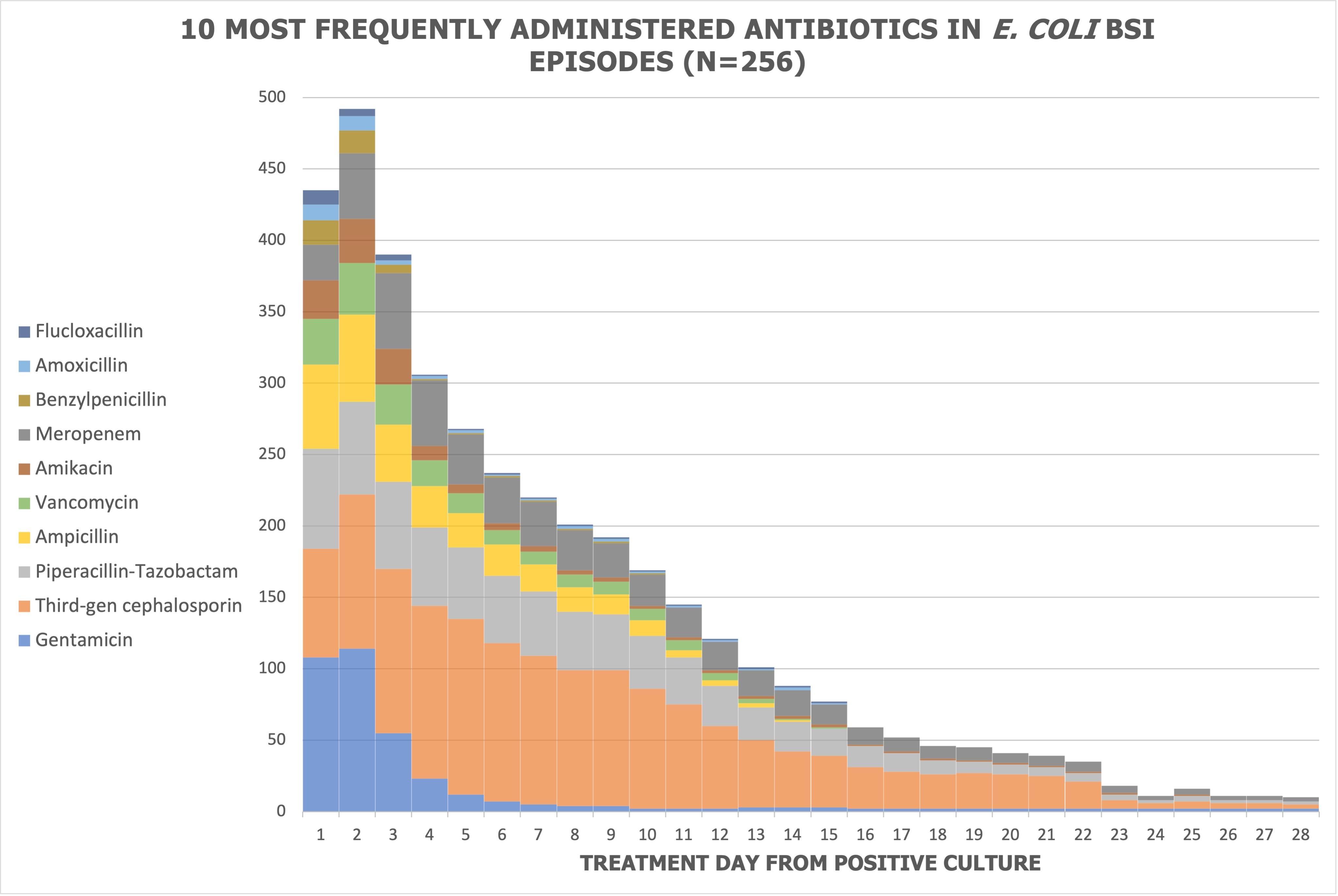

Supplement: ciae341_Supplementary_Data [file ciae341_supplementary_data.docx]
